# Chicken scFvs with an Artificial Cysteine for Site-Directed Conjugation

**DOI:** 10.1371/journal.pone.0146907

**Published:** 2016-01-14

**Authors:** Aerin Yoon, Jung Won Shin, Soohyun Kim, Hyori Kim, Junho Chung

**Affiliations:** 1 Department of Biochemistry and Molecular Biology, Seoul National University College of Medicine, Seoul National University, Seoul, South Korea; 2 Cancer Research Institute, Seoul National University College of Medicine, Seoul National University, Seoul, South Korea; University of Edinburgh, UNITED KINGDOM

## Abstract

For the site-directed conjugation of chemicals and radioisotopes to the chicken-derived single-chain variable fragment (scFv), we investigated amino acid residues replaceable with cysteine. By replacing each amino acid of the 157 chicken variable region framework residues (FR, 82 residues on V_H_ and 75 on V_L_) with cysteine, 157 artificial cysteine mutants were generated and characterized. At least 27 residues on V_L_ and 37 on V_H_ could be replaced with cysteine while retaining the binding activity of the original scFv. We prepared three V_L_ (L5, L6 and L7) and two V_H_ (H13 and H16) mutants as scFv-C_kappa_ fusion proteins and showed that PEG-conjugation to the sulfhydryl group of the artificial cysteine was achievable in all five mutants. Because the charge around the cysteine residue affects the *in vivo* stability of thiol-maleimide conjugation, we prepared 16 charge-variant artificial cysteine mutants by replacing the flanking residues of H13 with charged amino acids and determined that the binding activity was not affected in any of the mutants except one. We prepared four charge-variant H13 artificial cysteine mutants (RCK, DCE, ECD and ECE) as scFv-C_kappa_ fusion proteins and confirmed that the reactivity of the sulfhydryl group on cysteine is active and their binding activity is retained after the conjugation process.

## Introduction

Antibodies have been conjugated to chemical compounds for various purposes. Antibodies conjugated to enzymes are widely used in enzyme immunoassays or immunoblot analysis. Fluorescent dye-conjugated antibodies have applications in flow cytometric analysis, fluorescence immunoassays and fluorescence microscopy. For immunoaffinity purification, antibody-conjugated gels or magnetic beads are commonly used. Antibodies have also been conjugated to radioisotopes for use in radioimmunoassays, radioimmunoimaging and radioimmunotherapy. For clinical use, a technetium (99mTc)-labeled anti-CEA antibody (arcitumomab) is available for the detection of CEA-expressing tumors (CEA-scan) [1]. Radiolabeled anti-CD20 antibodies are used for the treatment of CD-20-expressing lymphoma and leukemia [2]. Antibody-drug conjugates (ADCs) have recently become available for the treatment of cancers. Two ADCs, trastuzumab emtansine (T-DM1, Kadcyla) and brentuximab vedotin (Adcetris), have been approved for the treatment of human epidermal growth factor receptor-2 (HER2)-positive metastatic and recurrent breast cancer and lymphoma, respectively [3].

Tyrosines, ε-amino acid chains of lysines, the carboxyl side chain of aspartic and glutamic acids and inter-chain disulfide bonds are frequently adopted as the functional residues for chemical cross-linking of an antibody to chemicals [4]. These covalent modifications require alkylation of tyrosines, acylation of lysine, amidation of carboxylates and reduction of cysteine to generate sulfhydryl groups [4, 5]. All these modifications occur randomly, which frequently impairs the antigen-binding activity of the antibody via the involvement of amino acids directly interacting with the antigen, or indirectly via conformational changes of the antibody after conjugation [6, 7]. To overcome this hurdle, site-specific conjugation using an artificial cysteine residue was introduced [6]. The 114th residue in the C_H_1 domain and the 442nd residue in C_H_3 have been successfully replaced with cysteine and used for cross-linking [6, 8–10].

The recent success of the chimeric antigen receptor T-cell therapy dramatically showed the potential of the single-chain variable fragment (scFv) in the clinical setting and described the need for more careful validation of the scFv, especially in the *in vivo* environment [11]. Radioimmuno positron emission tomography is an ideal tool for evaluating the specificity of the scFv, which can be achieved using radiolabeled scFv. To apply the chemistry developed for the cysteine-specific conjugation of IgG to scFv [12], it is essential to gain information about which residues can be switched to cysteine without affecting the affinity or increasing their aggregation tendency.

In this study we selected a chicken scFv as a model molecule, because there is only one chicken V_H_ and V_L_ gene and all the chicken scFvs share the same framework residues with the exception of occasional pseudogene usage [13]. We prepared a total of 157 artificial cysteine-switched scFv-displaying phages and tested their reactivity to the antigen. Among the positive clones, we selected five artificial cysteine-mutant scFvs, expressed them using a eukaryotic expression system and tested their binding activity. Furthermore, because the chemical and structural properties of the neighboring conjugation sites could potentially influence the stability of the antibody conjugate [14], we introduced mutations in the flanking residues and tested their effect on the binding activity. Finally, we prepared four flanking residue-switched charge-variant artificial cysteine mutants and confirmed that the binding activity of the mutant scFv was maintained after the chemical conjugation procedure.

## Materials and Methods

### Generation of the artificial cysteine mutants

Genes encoding artificial cysteine-mutant scFvs replacing each residue with cysteine between L4 and L22 and between H103 and H115 were generated by a single-step PCR using primers encoding the TGT sequence at the target nucleotides (S1 Table) and the gene encoding anti-prostate specific antigen (PSA) antibody as a template (GenBank accession numbers of VL and VH: KP764766 and KP764767, respectively). The PCR conditions were as follows: preliminary denaturation at 95°C for 5 min, followed by 30 cycles of 30 s at 95°C, 30 s at 60°C and 30 s at 72°C. The reaction ended with 5 min at 72°C. Genes encoding other artificial cysteine mutants were generated by two-step PCR. In the first PCR step, fragment 2 was generated containing the mutated region and fragment 1 was generated encoding the remainder of the scFv gene with an 18-nucleotide overlap. The PCR conditions were the same as described above. In the second overlap PCR step, the two gene fragments were linked to form a construct encoding the complete scFv gene. The PCR conditions were as follows: preliminary denaturation at 95°C for 5 min, followed by 25 cycles of 15 s at 95°C, 15 s at 56°C and 2 min at 72°C. The reaction ended with 10 min at 72°C. The primers were designed to add SfiI restriction sites at both ends of the gene. After restriction digestion with SfiI, the scFv genes were cloned into the pComb3XSS phagemid vector, as described previously [15]. The ligation products were transformed into E.coli ER2738 competent cells to generate 157 artificial cysteine-mutant scFv-displaying phage clones.

Charge-variant artificial cysteine-mutant scFvs were generated following the same procedure described above using primers to replace the flanking residues of the artificial H13 cysteine with lysine (AAG), arginine (CCT), aspartic acid (GAT), or glutamic acid (GAA) (S2 Table).

### Phage enzyme immunoassay

The scFv-displaying phages were rescued from titer plates after the transformation and subjected to a phage enzyme immunoassay as described previously [15]. The microtiter plates (half-area clear polystyrene; Corning, NY, USA) were coated with recombinant human Fc-tagged PSA by adding 20 μμl of the protein dissolved in phosphate-buffered saline (PBS) (5 μg/ml) to each well, followed by overnight incubation at 4°C. After blocking with 3% bovine serum albumin (BSA) dissolved in PBS (w/v, PBS-B), the plate was then sequentially incubated with scFv-displaying phages in the culture supernatant, horseradish peroxidase (HRP)-conjugated mouse anti-M13 monoclonal antibody (GE Healthcare, Pittsburg, PA, USA) in PBS-B and then finally with 2,2’-Azinobis [3-ethylbenzothiazoline-6-sulfonic acid]-diammonium salt (ABTS) substrate solutions (Amnesco LLC, Solon, OH, USA) with intermittent washing using 0.05% Tween-20 in PBS (PBST). After incubating the plates at 37°C for 10 min, the optical density was measured at 405 nm using a microtiter plate reader (Labsystems AiG SL, Barcelona, Spain).

### Expression of scFv-C_kappa_ fusion protein and conjugation

The genes encoding artificial cysteine-mutant scFvs (L5, L6, L7, H13 and H16 residues, Kabat numbering) and charge-variant artificial H13 cysteine mutant (RCK, DCE, ECD, ECE) were excised from pComb3X phagemid DNA with *Sfi*I restriction digestion, purified by 1% agarose gel electrophoresis and gel purification (Qiagen, Hilden, Germany) and cloned into an expression vector with the expression cassette for a scFv-C_kappa_ fusion protein that we reported earlier [16]. The expression vectors were transfected into human embryonic kidney (HEK293F, Invitrogen, Grand Island, NY, USA) cells, using polyethylenimine (Polysciences, Warrington, PA, USA) [17]. The transfected cells were cultured in FreeStyle 293 expression medium (Invitrogen) in a 37°C CO_2_ incubator for 6 days as described previously [18]. The scFv-C_kappa_ fusion protein in the culture supernatant was purified by affinity chromatography using KappaSelect resin (GE Healthcare) following the guidelines provided by the supplier. Conjugation of scFv-C_kappa_ fusion proteins to PEG or cotinine was performed as reported previously [9, 19]. Briefly, the fusion protein was fully reduced by adding ten-fold molar excess of TCEP-HCl (Pierce Chemical, Rockford, IL, USA) and incubating 1 h at 37°C. Following dia-filtration using Amicon-ultra centrifugal filters (Merck Millipore, Darmstadt, Germany), the proteins were incubated for 3 h at 25°C with dehydroascorbic acid (Sigma-Aldrich, St. Louis, MO, USA) at a three-fold molar excess over the TCEP-HCl concentration. The maleimide-linked PEG (Nof Corporation, Tokyo, Japan) or GMBS-coupled cotinine (Peptron, Daejeon, South Korea) reagents (ten-fold molar protein) were incubated overnight with the activated proteins at 4°C. The reactants were filtered using the Amicon-ultra centrifugal filter (Merck Millipore) and washed five times with PBS to remove any remaining reagents.

### SDS-polyacrylamide gel electrophoresis and staining

The PEG conjugated scFv-C_kappa_ fusion proteins were analyzed by SDS-polyacrylamide gel electrophoresis. Two replicates containing 10 μg of fusion protein were dissolved in LDS sample buffer (Invitrogen) without reducing agents, boiled for 5 min and electrophoresed on 4–12% (w/v) Tris-glycine gradient gels (Novex^®^ NuPAGE^®^, Invitrogen). One of the gels was stained with 0.1% Coomassie blue R250 in 10% acetic acid, 50% methanol and 40% distilled water for 30 min and de-stained with 10% acetic acid/50% methanol for 1 hr. The other gel was washed in distilled water for 15 min at room temperature, then soaked in 5% barium chloride solution for 15 min and another 10 min in 0.1 M iodine solution. The stained PEGylated protein appeared as an orange brown color. The solution was replaced with distilled water for background washing.

### Enzyme immunoassay

Recombinant human Fc-tagged PSA was dissolved in 20 μl of 0.1 M NaHCO_3_ (pH 8.6), added to microtiter plate wells and incubated overnight at 4°C. The plates were washed three times with PBST and incubated for 1 h at 37°C with PBS-B. After washing the plates with PBST, 250 ng of cotinine conjugated scFv-C_kappa_ fusion protein in 50 μl of PBS-B was added to each well. After incubation for 1 h at 37°C and washing with PBST, the plates were incubated with HRP-conjugated anti-human C_kappa_ antibody (Thermo Fisher Scientific; Waltham, MA, USA) diluted 5,000-fold in PBS-B or 0.5 μg of HRP-conjugated anti-cotinine IgG_1_ dissolved in 50 μl PBS-B. After washing with PBST, TMB substrate solution (GeneDEPOT, Barker, TX, USA) was added to each well. The optical density was measured at 450 nm with a microtiter-plate reader (Labsystems AiG S.L.).

### Statistical analysis

The statistical analysis was performed using Student’s t-tests employing SigmaPlot 10.0 software (SPSS; Chicago, IL, USA).

## Results

### Characterization of the artificial cysteine mutants

Because chicken has only one functional V_H_ and V_L_ gene, all the chicken scFvs share the same framework residues [20]. Once the residues replaceable with cysteine were determined using one chicken scFv, the data would be more easily applied to other chicken scFv molecules than to the scFvs of other animal species with multiple V_H_ and V_L_ genes. In this context we selected a chicken rather than using other model animal species.

**Fig 1:**
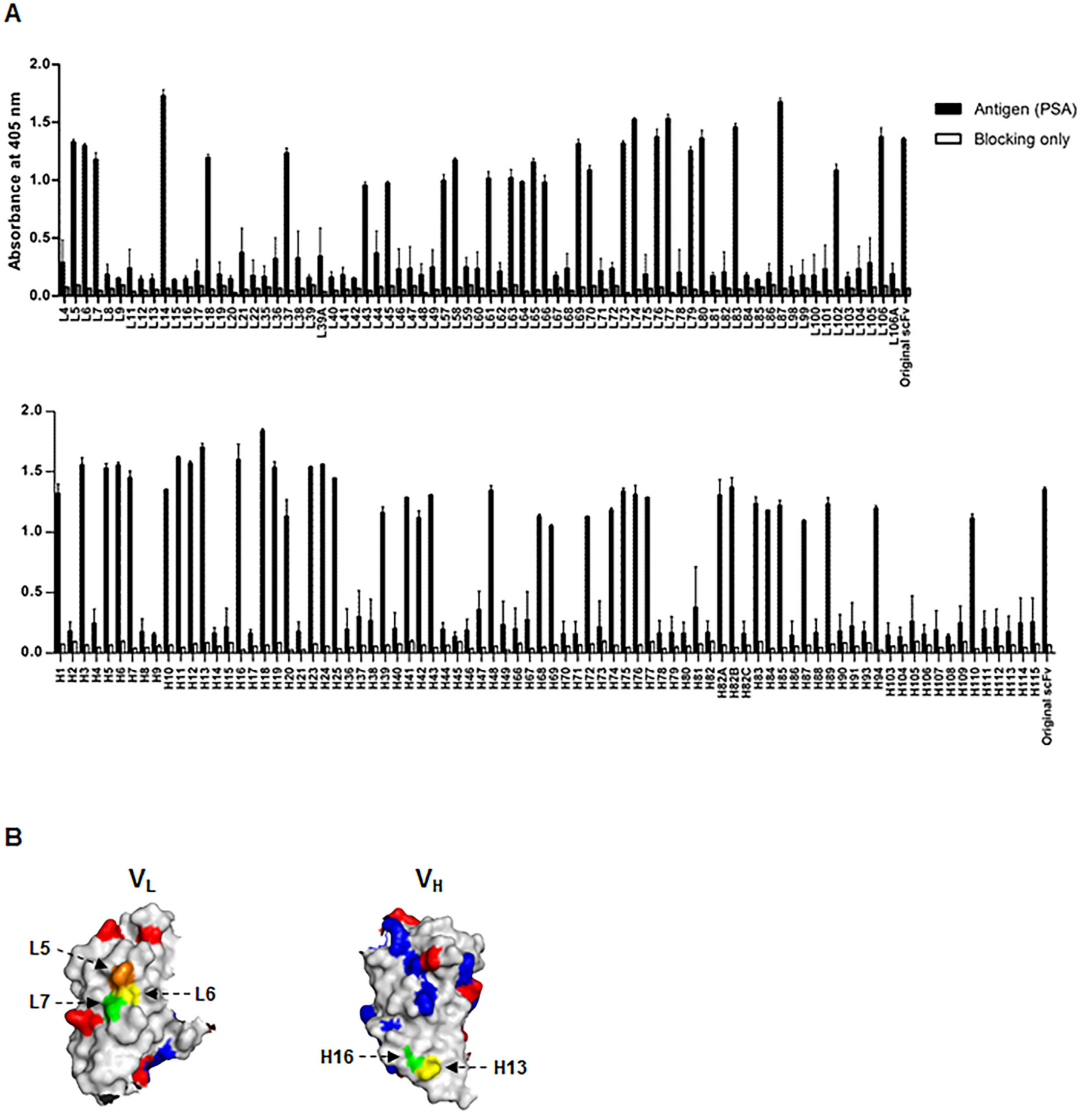
Reactivity of scFvs displayed on phage with a point mutation. The binding activity of 157 cysteine-mutants in the form of scFv-pIII displayed on phage was tested using recombinant human Fc-tagged PSA coated on a microtiter plate and an HRP-conjugated anti-M13 antibody. The assays were performed in duplicates. (A) The optical density of the artificial cysteine-mutant scFvs’ retaining binding activity is shown in the graph. H and L indicate the V_H_ and V_L_ mutants, respectively. The number after H or L denotes the Kabat number. (B) The three-dimensional structure of the chicken antibody variable regions (PBD: 4GLR) was visualized by PyMOL 1.3 (http://www.pymol.org). The residues L5, L6 and L7 are marked by orange, yellow and green colors, respectively. The H13 and H1 residues are individually marked with yellow and green colors, respectively. Blue and red colors indicate positively charged R groups (K, R and H) and negatively charged R groups (D and E), respectively. All data represent the average ± standard derivation (SD) of triplicate experiments.

By replacing each amino acid of the 157 FR residues (82 V_H_ and 75 V_L_) with cysteine, a total of 157 artificial cysteine-mutant scFvs were prepared in the form of a scFv-pIII fusion protein displayed on the phage surface. Then, the reactivity of artificial cysteine-mutant scFvs toward the prostate-specific antigen (PSA) was tested in a phage enzyme immunoassay. The artificial cysteine mutant scFvs could be classified into either positive or negative groups with significantly different averages (p < 0.05) when the cut-off value of the average absorbance was set at 0.9 (S1 Fig). Sixty-four artificial cysteine mutant scFvs were classified into the positive group (37 V_H_ and 27 V_L_) (Fig 1A). Out of 64 mutants, we selected three V_L_ (L5, L6 and L7) and two V_H_ (H13 and H16) artificial cysteine-mutants for further studies, because these residues were located away from the binding site, not in the interface between V_H_ and V_L_ and on the loop structure in the publically available chicken antibody three-dimensional structure (PDB: 4GLR; Fig 1B) [21]. L77 has already been reported to be replaceable with cysteine and therefore has not been included in this study (Patent No. US 7,521,541 B2 and EP2579897 A1).

### PEGylation of artificial cysteine-mutant scFv-C_kappa_ fusion proteins using thiol-maleimide chemistry

**Fig 2:**
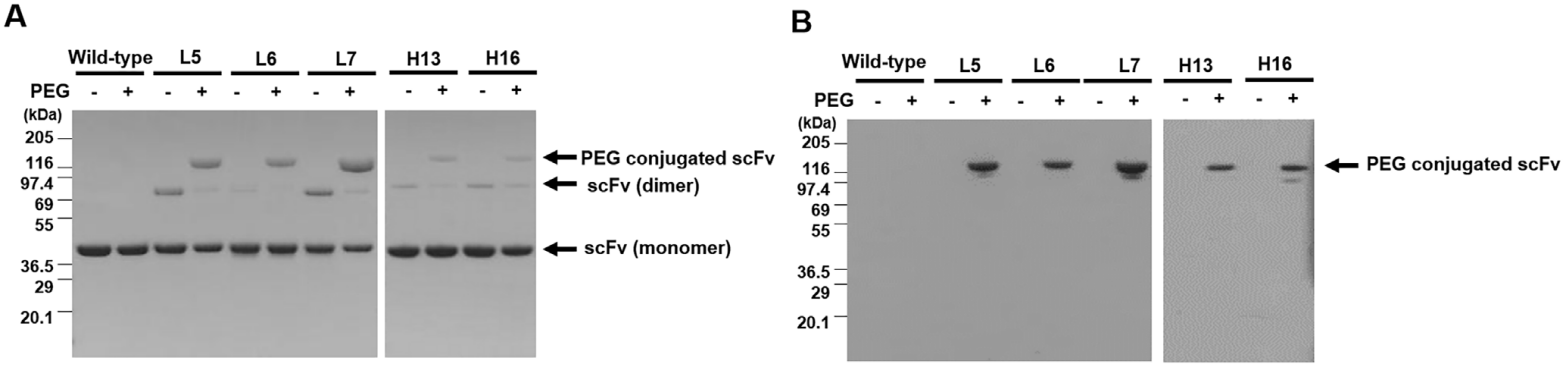
SDS-polyacrylamide gel electrophoresis analysis of the artificial cysteine mutant scFv-C_kappa_ fusion protein PEG conjugates. Artificial cysteine-mutant scFv-C_kappa_ fusion protein PEG conjugates were subjected to 4–12% (w/v) SDS-polyacrylamide gel electrophoresis without the addition of a reducing agent. After electrophoresis, the gel was stained with Coomassie Blue (A) or barium iodide (I_2_) (B). An artificial cysteine-mutant scFv-C_kappa_ fusion protein not conjugated with PEG and the original scFv-C_kappa_ fusion protein were used as controls.

Five artificial cysteine mutant scFvs fused with C_kappa_ were expressed and purified using an eukaryotic expression system. Then, their binding activity toward PSA was confirmed once more (data not shown). After conjugation with the maleimide functionalized PEG, the fusion proteins were analyzed by SDS-polyacrylamide gel electrophoresis and Coomassie staining or barium iodine (I_2_) staining, which can visualize both the protein-bound and free PEG. After Coomassie staining, three major bands with the molecular weights of 116, 80 and 40 kDa were visualized (Fig 2A). In the barium iodine (I_2_) staining, only one band with a molecular weight of 116 kDa was visualized, which proved that the scFv-C_kappa_ fusion protein-PEG conjugate was successfully formed in all artificial cysteine-mutant scFvs (Fig 2B). The protein bands with molecular weight 80 and 40 are expected to be a dimer and monomer of the artificial cysteine scFv-C_kappa_ fusion protein not reacted with PEG, respectively.

### Effect of substituting the flanking residues of artificial cysteine with charged amino acids

**Fig 3:**
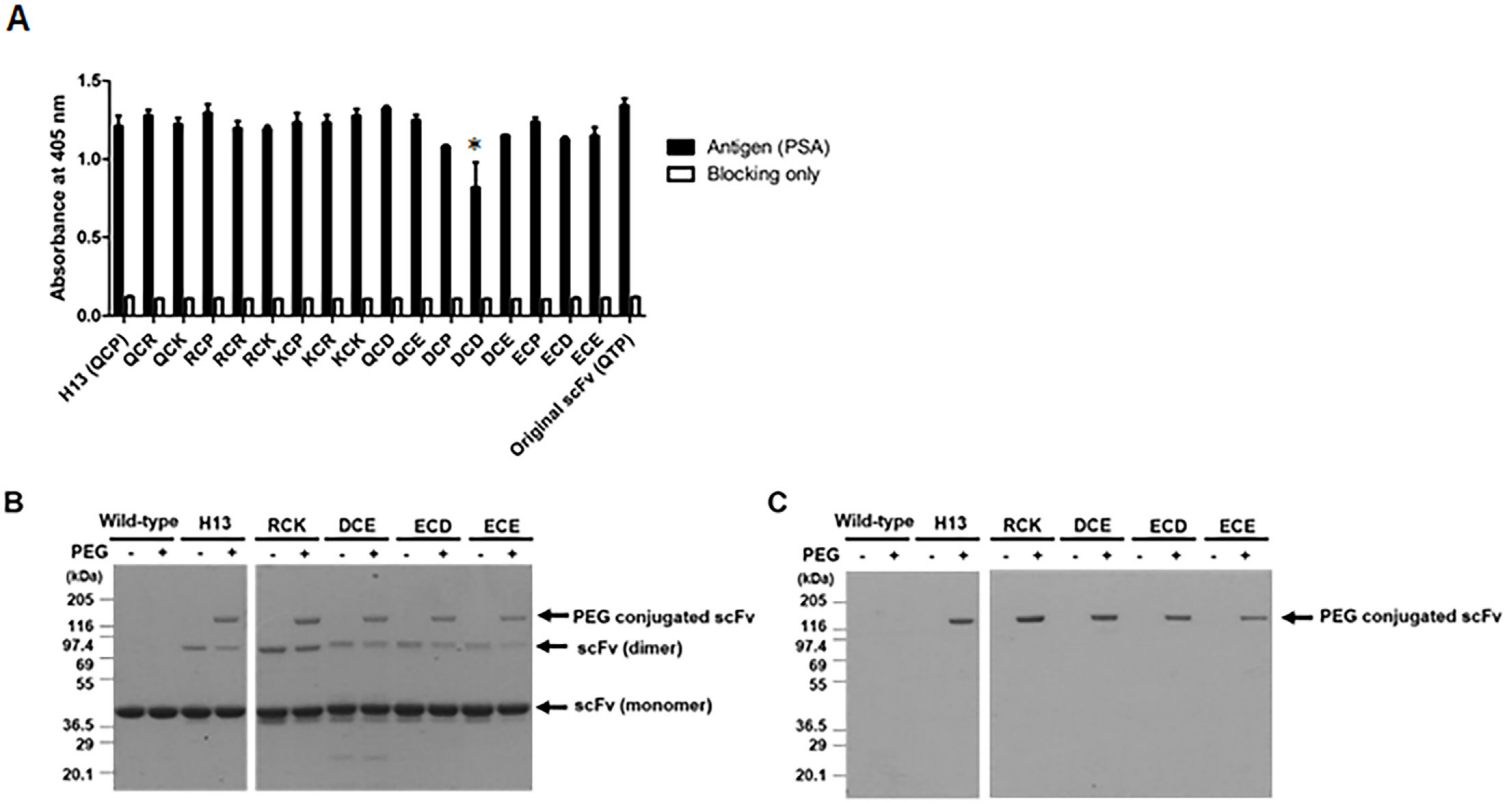
Analysis of the charge-variant artificial cysteine-mutant scFvs. (A) Sixteen charge-variant artificial cysteine-mutant scFvs were prepared as scFv-PIII fusion proteins displayed on phage. Their binding activity was tested in a phage enzyme immunoassay using recombinant human Fc-tagged PSA coated on a microtiter plate and an HRP-conjugated anti-M13 antibody. The assays were performed in triplicates. (B) Four charge-variant artificial H13 cysteine-mutant scFvs were prepared as scFv-C_kappa_ fusion proteins, conjugated with PEG and subjected to 4–12% (w/v) SDS-polyacrylamide gel electrophoresis without the addition of a reducing agent. After electrophoresis, the gel was stained with Coomassie Blue. (C) The gel prepared in parallel was subjected to barium iodide (I_2_) staining. A charge-variant artificial cysteine-mutant scFv-C_kappa_ fusion protein not conjugated with PEG and the original scFv-C_kappa_ fusion protein were used as controls. All data represent the average ± standard derivation (SD) of triplicate experiments. *, p < 0.05 (vs. H13) as determined by student’s t-test.

Because the charges around the artificial cysteine on the antibody affect the *in vivo* stability of the conjugate [14], we tested the effects of substituting the flanking residues with charged amino acids on the binding activity of the artificial cysteine-mutant scFv. Sixteen charge-variant artificial cysteine-mutant scFvs, in which the flanking residues of cysteine at the H13 position were replaced by either positively charged R groups (lysine and arginine) or negatively charged R groups (aspartic acid and glutamic acid), were prepared in the form of scFv-pIII fusion proteins displayed on the surface of the phage and their binding activity was tested in the phage enzyme immunoassay. In the case of H13, none of the artificial cysteine-mutant scFvs showed a statistically different average absorbance from that of artificial cysteine mutant scFv (H13 (QCP)) (p > 0.05), with the exception of the artificial cysteine-mutant scFv with the sequence DCD (Fig 3A). In the H16, L5, L6 and L7 positions, charge-variant artificial cysteine-mutant scFv clones with binding activity were observed less frequently (S2 Fig).

We overexpressed and purified four charge-variant artificial H13 cysteine-mutant scFvs (RCK, DCE, ECD and ECE) as scFv-C_kappa_ fusion proteins. The fusion proteins were subjected to conjugation with maleimide functionalized polyPEG and analyzed by an acrylamide gel electrophoresis. Coomassie and barium iodine (I_2_) staining revealed that PEGylated scFvs were formed in H13, RCK, DEC, ECD and ECE charge-variant artificial cysteine-mutant scFvs as expected (Fig 3B and 3C).

### Conjugation of cotinine to charge-variant artificial cysteine-mutant scFvs

**Fig 4:**
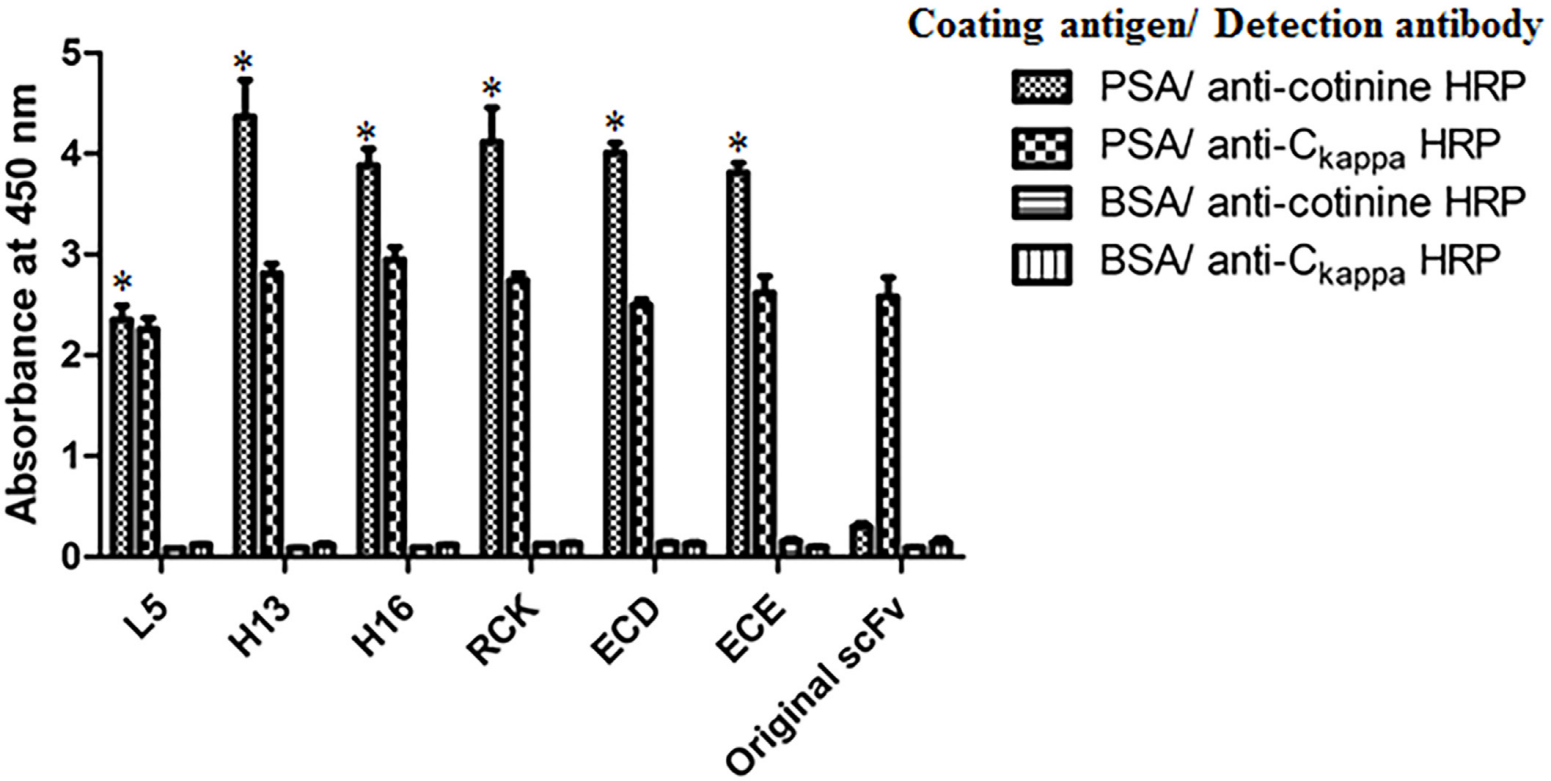
Binding activity of a charge-variant artificial cysteine-mutant scFv-C_kappa_ fusion protein cotinine conjugate. The binding activity of four charge-variant artificial cysteine-mutant scFv-C_kappa_ fusion protein cotinine conjugates was tested in an enzyme immunoassay using recombinant Fc-tagged PSA coated on a microtiter plate and an anti-cotinine antibody conjugated with HRP. In parallel, an HRP-conjugated anti-C_kappa_ antibody was used to determine the amount of scFv- C_kappa_ fusion protein bound to the plate. Original scFv-C_kappa_ fusion protein was used as a control protein. All data represent the average ± standard derivation (SD) of triplicate experiments. *, p < 0.05 (vs. original scFv) as determined by student’s t-test.

To confirm whether the conjugation process affects the binding activity of a charge-variant artificial cysteine-mutant scFv, we conjugated the scFv-C_kappa_ fusion protein to cotinine using GMBS-coupled cotinine (GMBS-G-G-G-Cit-V-cotinine). Then, the binding activity of the fusion protein cotinine conjugates was tested in an enzyme immunoassay using a microtiter plate coated with recombinant human Fc-tagged PSA and anti-cotinine antibody conjugated with HRP. All the fusion protein cotinine conjugates reacted with PSA and anti-cotinine antibody simultaneously (Fig 4), which proved that the charge-variant artificial cysteine-mutant scFv still had binding activity even after the conjugation process and that the conjugated cotinine is exposed to outside, allowing it to be bound by anti-cotinine antibody conjugated with horseradish peroxidase.

## Discussion

Antibodies bind a target molecule through the antigen binding sites composed of two variable regions (V_H_ and V_L_). The fragment crystallizable (Fc) domain binds to Fc receptor and complement to exert its effector function. The binding of Fc to neonatal Fc receptors provides a long half-life of antibodies [22, 23]. Full-length IgG have been a standard format for therapeutic, diagnostic, or research purposes.

However, there are situations in which an Fc-mediated effect is undesirable [24, 25]. For example, the antibodies could have an undesired distribution to organs like the bone marrow and spleen via their interaction with cells that have Fc receptors. Therefore, when the antibody is used only as a guiding molecule for liposomes, scFv is usually the preferred format. An anti-HER2 scFv conjugated with liposomal doxorubicin is currently under phase II clinical trial [26]. A long serum half-life is also a disadvantage in imaging applications, where faster clearance is required in order to limit exposure to radionucleotide molecules [27].

The scFv consists of V_H_ and V_L_ joined together by a flexible peptide linker [28, 29]. Compared to full antibodies, scFv has unique pharmacokinetic properties. For instance, the scFv can penetrate more rapidly into tumors compared to an intact antibody [30, 31]. Due to its small size and lack of interaction with neonatal Fc receptors, scFv is cleared from blood more rapidly. These properties are quite beneficial, especially when scFv is conjugated with radioisotopes and cytotoxic drugs, because the exposure of healthy tissues can be minimized [32]. In the development of bispecific antibodies, two scFvs are commonly adopted as a platform [33]. A bispecific scFv against CD3 and CD19, known as blinatumomab (Blincyto), is approved for the treatment of Philadelphia chromosome-negative acute lymphoblastic leukemia [34]. Additionally, a bispecific scFv against CEA and CD3 (MEDI-565, AMG-211) is under phase II clinical trial [26]. A tetravalent, bispecific antibody made of scFvs against CD30 and CD16A (AFM13) is also under phase II clinical trial [26].

The recent success of chimeric antigen receptor (CAR) T-cell therapy dramatically increased the use of scFv in clinical settings [35]. The injection of CAR T-cell elicits a cytokine storm syndrome in some patients [36] and sometimes causes a serious adverse event [37]. Whether the cytokine storm syndrome is elicited in on-target or off-target binding of the scFv is not certain. Therefore, careful validation of scFv specificity is critical; this can be achieved by radioimmuno positron emission tomography using a radio-labeled scFv [38, 39].

Historically, the labeling of scFv was performed using fusion tags, providing the sites for the chemical reaction [40, 41], because it was not certain whether the cysteine residue-dependent site-specific conjugation chemistry developed for the attachment of the full IgG molecule to scFv is applicable to scFv. To achieve this, the residues replaceable with cysteine without hindering the binding activity or solubility of scFv should be screened out and the binding activity of mutant scFv should not be affected by the conjugation process. For *in vivo* applications, the chemical link between the scFv and conjugated chemicals should be stable for a reasonable period of time. All of these issues were addressed in this study. A chicken scFv was selected as a model molecule. Because there is only one chicken V_H_ and V_L_ gene, all the chicken scFvs share the same framework residues with the exception of occasional pseudogene usage [13]. We screened 157 framework region residues (82 V_H_ and 75 V_L_) except for the four cysteines forming the intra-domain disulfide bonds. Surprisingly, at least 27 residues in V_L_ and 37 in V_H_ could be replaced with cysteine while retaining the binding activity in the form of an scFv-pIII fusion molecule displayed on a phage (Fig 1A) [15]. We focused on three V_L_ (L5, L6 and L7) and two V_H_ (H13 and H16) mutants, because they showed partial solvent-accessibility in the three-dimensional structure of a chicken antibody whose three-dimensional structure is publically available (PBD: 4GLR; Fig 1B) [21] and because these residues were away from the antigen binding sites. Although the degree of exposure of an artificial cysteine residue to water is critical for efficient conjugation, partial solvent-accessibility is advantageous for longer *in vivo* stability of the conjugate [14]. The artificial cysteines’ sulfhydryl groups on all five artificial cysteine-mutant scFvs showed reactivity to maleimide functionalized PEG after a conventional reduction and oxidation procedure (Fig 2) [9, 19]. Because the charges around the cysteine are critical for the *in vivo* stability of the conjugate [14], we tested the effect of changing the flanking residues of an artificial cysteine using an artificial H13 cysteine-mutant scFv. Interestingly, the charge-variant artificial H13 cysteine-mutant scFvs in the form of scFv-pIII fusion proteins retained binding activity, with the exception of one with the sequence of DCD (Fig 3A). We prepared four charge-variant artificial cysteine H13 mutant scFvs (RCK, DCE, ECD and ECE) as scFv-C_kappa_ fusion proteins and confirmed that the sulfhydryl group on the artificial cysteines is reactive and that their binding activity was retained after the conjugation process. The binding activity of the L5 mutant scFv measured using anti-cotinine antibody-HRP conjugate was relatively lower than that of the other artificial cysteine-mutant scFvs. One of the reasons may be steric hindrance around the cotinine. We used a linker that is 4 amino acids in length, which might not be long enough to allow the conjugated cotinine to be free of steric hindrance.

We and another group previously observed that the structure of the chicken scFv is very much plastic and can harbor a certain range of mutations without losing binding activity and solubility [20] (manuscript under revision). In addition to these observations, our study claims that the conjugation of chemicals to scFv in a site-directed manner can be achieved without hindering the binding activity and solubility, which opens a new way of labeling a scFv with an isotope or chemicals.

## Supporting Information

S1 Fig Binding activity of positive and negative cysteine mutant scFvs. The artificial cysteine mutant scFvs were classified into either positive or negative groups based on an average absorbance cut-off value of 0.9. The average absorbance and standard deviation of the original scFv was 1.35 and 0.019, respectively. The median absorbance of the positive and negative groups was 1.3 and 0.19, respectively. The average absorbance was significantly different between the two groups (p < 0.05). Boxes span the interquartile range, the line within each box denotes the median, and whiskers indicate the minimum and maximum values.
(TIF)

S2 Fig Reactivity of the charge-variant artificial cysteine-mutant scFvs. The binding activity of charge-variant artificial cysteine-mutants in the form of scFv-pIII fusion proteins displayed on phage was tested. Eight (A) positive and (B) negative charge-variant artificial cysteine-mutant scFvs of positions L5, L6, L7, H13 and H16 were tested in a phage enzyme immunoassay, with recombinant human Fc-tagged PSA protein coated on a microtiter plate and an HRP-conjugated anti-M13 antibody, respectively. *, p < 0.05 (vs. each cysteine-mutants) as determined by student’s t-test.
(TIF)

S1 Table PCR primer list for generation of the artificial cysteine-mutants. (DOCX)

S2 Table PCR primer list for generation of charge-variant artificial cysteine-mutant. (DOCX)
