# Supplementary figures and images for: Chicken scFvs with an Artificial Cysteine for Site-Directed Conjugation
Source: PLoS One. 2016 Jan 14;11(1):e0146907. doi: 10.1371/journal.pone.0146907 (PMC4713166; doi:10.1371/journal.pone.0146907)

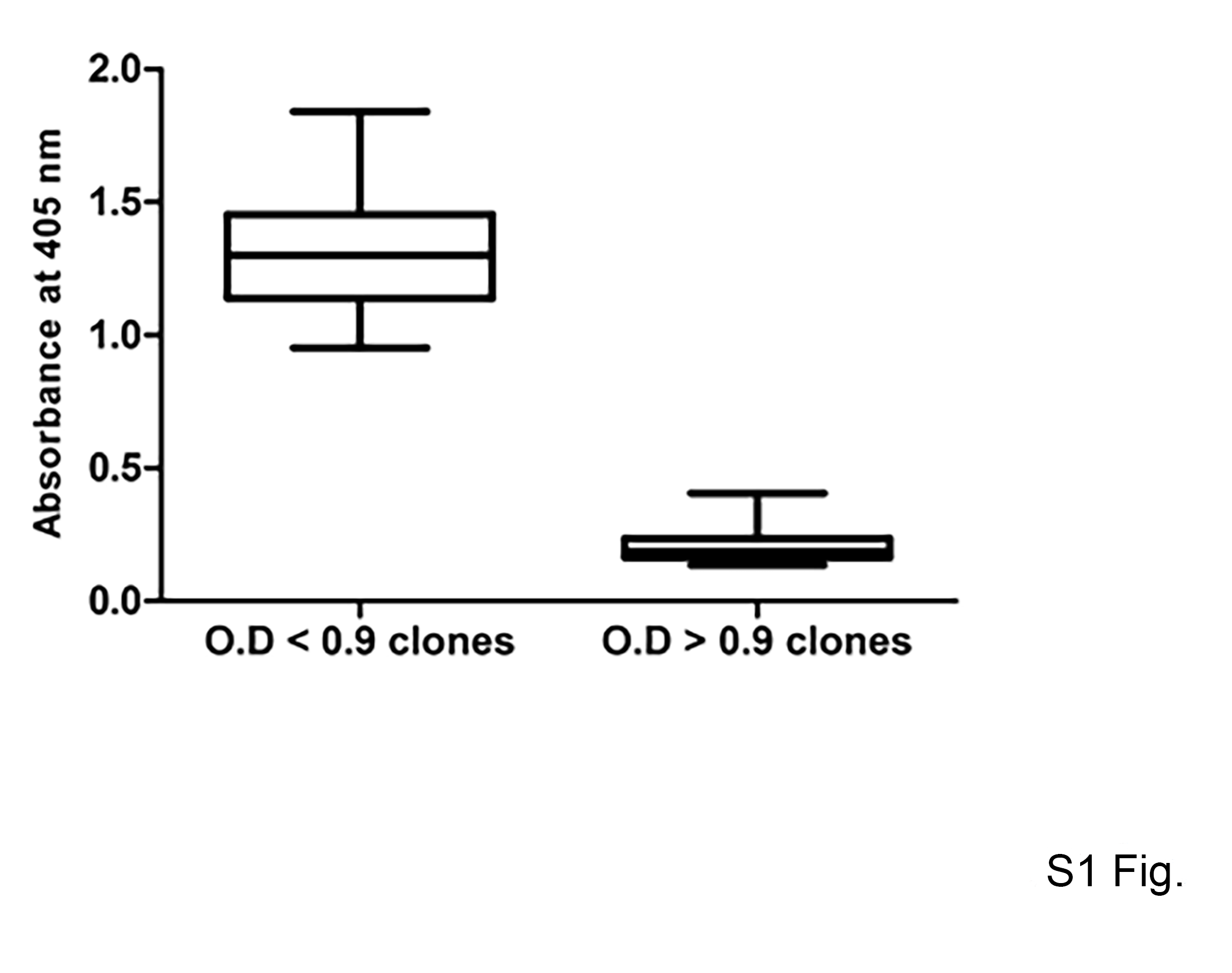

Supplement: S1 Fig — The artificial cysteine mutant scFvs were classified into either positive or negative groups based on an average absorbance cut-off value of 0.9. The average absorbance and standard deviation of the original scFv was 1.35 and 0.019, respectively. The median absorbance of the positive and negative groups was 1.3 and 0.19, respectively. The average absorbance was significantly different between the two groups (p < 0.05). Boxes span the interquartile range, the line within each box denotes the median, and whiskers indicate the minimum and maximum values. (TIF) [file pone.0146907.s001.tif]

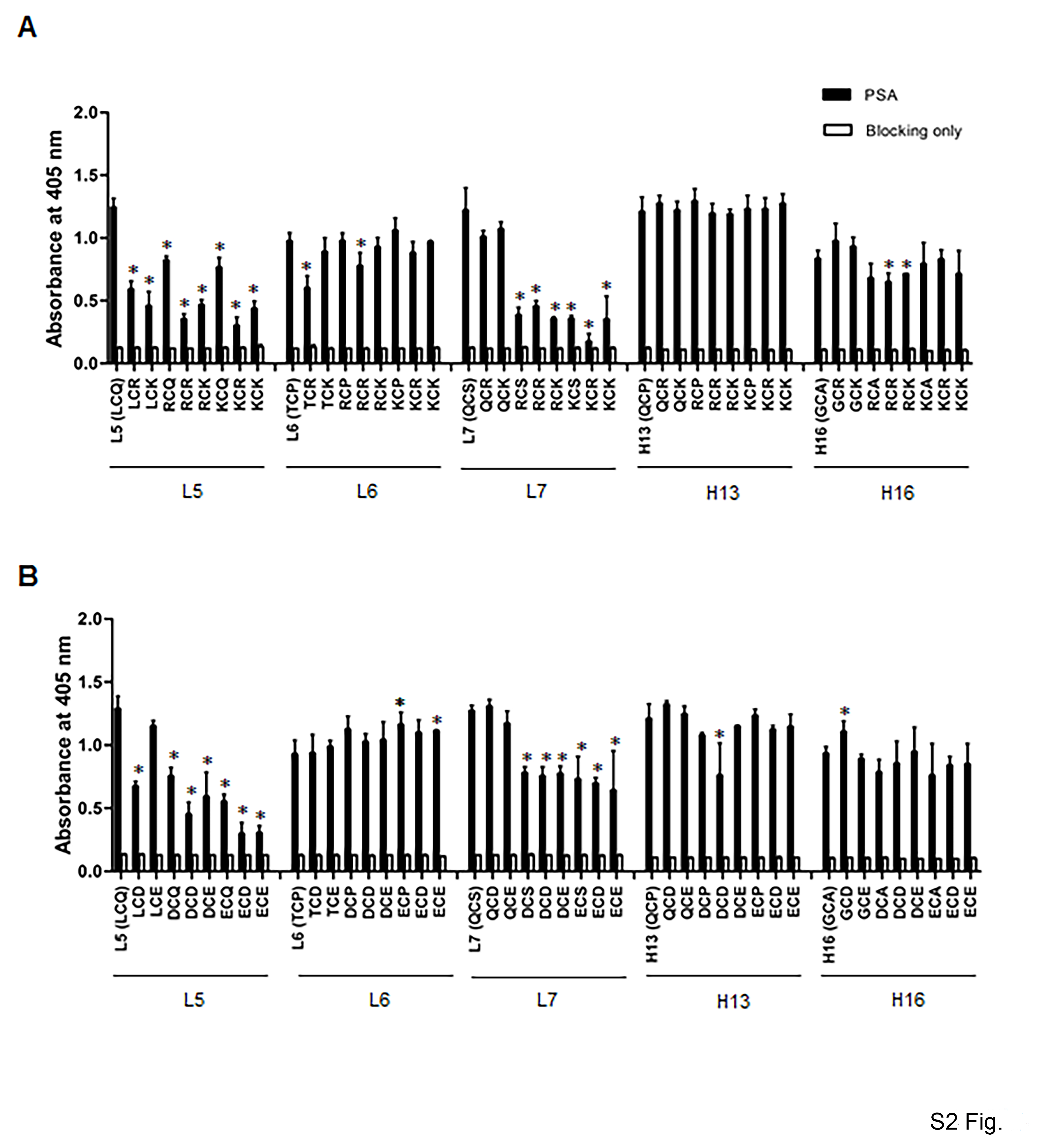

Supplement: S2 Fig — The binding activity of charge-variant artificial cysteine-mutants in the form of scFv-pIII fusion proteins displayed on phage was tested. Eight (A) positive and (B) negative charge-variant artificial cysteine-mutant scFvs of positions L5, L6, L7, H13 and H16 were tested in a phage enzyme immunoassay, with recombinant human Fc-tagged PSA protein coated on a microtiter plate and an HRP-conjugated anti-M13 antibody, respectively. *, p < 0.05 (vs. each cysteine-mutants) as determined by student’s t-test. (TIF) [file pone.0146907.s002.tif]
