# Supplementary material for: Chicken scFvs with an Artificial Cysteine for Site-Directed Conjugation
Source: PLoS One. 2016 Jan 14;11(1):e0146907. doi: 10.1371/journal.pone.0146907 (PMC4713166; doi:10.1371/journal.pone.0146907)
Supplement: S1 Table — (DOCX) [file pone.0146907.s003.docx]

S1 Table. PCR primer list for generation of the artificial cysteine-mutants

| **Kabat #** | **Original sequence** | **Fragment** | **Primer sequence** | |
| --- | --- | --- | --- | --- |
|  |  |  | **Forward (5` -> 3`)** | **Reverse (5` -> 3`)** |
| **L4** | **L** |  | GGCCCAGGCGGCC TGT ACT CAG CCG TCC TCG | GGC CGG CCT GGC CAC TAG TGG AGG AGA CGA TGA |
| **L5** | **T** |  | GGCCCAGGCGGCC CTG TGT CAG CCG TCC TCG GTG | GGC CGG CCT GGC CAC TAG TGG AGG AGA CGA TGA |
| **L6** | **Q** |  | GGCCCAGGCGGCC CTG ACT TGT CCG TCC TCG GTG TCA | GGC CGG CCT GGC CAC TAG TGG AGG AGA CGA TGA |
| **L7** | **P** |  | GGCCCAGGCGGCC CTG ACT CAG TGT TCC TCG GTG TCA GCA | GGC CGG CCT GGC CAC TAG TGG AGG AGA CGA TGA |
| **L8** | **S** |  | GGCCCAGGCGGCC CTG ACT CAG CCG TGT TCG GTG TCA GCA AAC | GGC CGG CCT GGC CAC TAG TGG AGG AGA CGA TGA |
| **L9** | **S** |  | GGCCCAGGCGGCC CTG ACT CAG CCG TCC TGT GTG TCA GCA AAC CTG | GGC CGG CCT GGC CAC TAG TGG AGG AGA CGA TGA |
| **L11** | **V** |  | GGCCCAGGCGGCC CTG ACT CAG CCG TCC TCG TGT TCA GCA AAC CTG GGA | GGC CGG CCT GGC CAC TAG TGG AGG AGA CGA TGA |
| **L12** | **S** |  | GGCCCAGGCGGCC CTG ACT CAG CCG TCC TCG GTG TGT GCA AAC CTG GGA GGA | GGC CGG CCT GGC CAC TAG TGG AGG AGA CGA TGA |
| **L13** | **A** |  | GGCCCAGGCGGCC CTG ACT CAG CCG TCC TCG GTG TCA TGT AAC CTG GGA GGA ACC | GGC CGG CCT GGC CAC TAG TGG AGG AGA CGA TGA |
| **L14** | **N** |  | GGCCCAGGCGGCC CTG ACT CAG CCG TCC TCG GTG TCA GCA TGT CTG GGA GGA ACC GTC | GGC CGG CCT GGC CAC TAG TGG AGG AGA CGA TGA |
| **L15** | **L** |  | GGCCCAGGCGGCC CTG ACT CAG CCG TCC TCG GTG TCA GCA AAC TGT GGA GGA ACC GTC AAG | GGC CGG CCT GGC CAC TAG TGG AGG AGA CGA TGA |
| **L16** | **G** |  | GGCCCAGGCGGCC CTG ACT CAG CCG TCC TCG GTG TCA GCA AAC CTG TGT GGA ACC GTC AAG ATC | GGC CGG CCT GGC CAC TAG TGG AGG AGA CGA TGA |
| **L17** | **G** |  | GGCCCAGGCGGCC CTG ACT CAG CCG TCC TCG GTG TCA GCA AAC CTG GGA TGT ACC GTC AAG ATC ACC | GGC CGG CCT GGC CAC TAG TGG AGG AGA CGA TGA |
| **L18** | **T** |  | GGCCCAGGCGGCC CTG ACT CAG CCG TCC TCG GTG TCA GCA AAC CTG GGA GGA TGT GTC AAG ATC ACC TGT | GGC CGG CCT GGC CAC TAG TGG AGG AGA CGA TGA |
| **L19** | **V** |  | GGCCCAGGCGGCC CTG ACT CAG CCG TCC TCG GTG TCA GCA AAC CTG GGA GGA ACC TGT AAG ATC ACC TGT TCC | GGC CGG CCT GGC CAC TAG TGG AGG AGA CGA TGA |
| **L20** | **K** |  | GGCCCAGGCGGCC CTG ACT CAG CCG TCC TCG GTG TCA GCA AAC CTG GGA GGA ACC GTC TGT ATC ACC TGT TCC GGG | GGC CGG CCT GGC CAC TAG TGG AGG AGA CGA TGA |
| **L21** | **I** |  | GGCCCAGGCGGCC CTG ACT CAG CCG TCC TCG GTG TCA GCA AAC CTG GGA GGA ACC GTC AAG TGT ACC TGT TCC GGG GGT | GGC CGG CCT GGC CAC TAG TGG AGG AGA CGA TGA |
| **L22** | **T** |  | GGCCCAGGCGGCC CTG ACT CAG CCG TCC TCG GTG TCA GCA AAC CTG GGA GGA ACC GTC AAG ATC TGT TGT TCC GGG GGT TAC | GGC CGG CCT GGC CAC TAG TGG AGG AGA CGA TGA |
|  |  |  |  |  |
| **L35** | **W** | fragment 1 | GGCCCAGGCGGCC CTG ACT CAG CCG TCC TCG | GCC GTA GTA GCT GTA ACC CCC |
|  |  | fragment 2 | GGG GGT TAC AGC TAC TAC GGC TGT TAT CAG CAG AAG TCT CCT | GGC CGG CCT GGC CAC TAG TGG AGG AGA CGA TGA |
|  |  | overlap | GGCCCAGGCGGCC CTG ACT CAG CCG TCC TCG | GGC CGG CCT GGC CAC TAG TGG AGG AGA CGA TGA |
| **L36** | **Y** | fragment 1 | GGCCCAGGCGGCC CTG ACT CAG CCG TCC TCG | ATA CCA GCC GTA GTA GCT GTA |
|  |  | fragment 2 | TAC AGC TAC TAC GGC TGG TAT TGT CAG AAG TCT CCT GGC AGT | GGC CGG CCT GGC CAC TAG TGG AGG AGA CGA TGA |
|  |  | overlap | GGCCCAGGCGGCC CTG ACT CAG CCG TCC TCG | GGC CGG CCT GGC CAC TAG TGG AGG AGA CGA TGA |
| **L37** | **Q** | fragment 1 | GGCCCAGGCGGCC CTG ACT CAG CCG TCC TCG | ATA CCA GCC GTA GTA GCT GTA |
|  |  | fragment 2 | TAC AGC TAC TAC GGC TGG TAT TGT CAG AAG TCT CCT GGC AGT | GGC CGG CCT GGC CAC TAG TGG AGG AGA CGA TGA |
|  |  | overlap | GGCCCAGGCGGCC CTG ACT CAG CCG TCC TCG | GGC CGG CCT GGC CAC TAG TGG AGG AGA CGA TGA |
| **L38** | **Q** | fragment 1 | GGCCCAGGCGGCC CTG ACT CAG CCG TCC TCG | CTG ATA CCA GCC GTA GTA GCT |
|  |  | fragment 2 | AGC TAC TAC GGC TGG TAT CAG TGT AAG TCT CCT GGC AGT GCC | GGC CGG CCT GGC CAC TAG TGG AGG AGA CGA TGA |
|  |  | overlap | GGCCCAGGCGGCC CTG ACT CAG CCG TCC TCG | GGC CGG CCT GGC CAC TAG TGG AGG AGA CGA TGA |
| **L39** | **K** | fragment 1 | GGCCCAGGCGGCC CTG ACT CAG CCG TCC TCG | CTG CTG ATA CCA GCC GTA GTA |
|  |  | fragment 2 | TAC TAC GGC TGG TAT CAG CAG TGT TCT CCT GGC AGT GCC CCT | GGC CGG CCT GGC CAC TAG TGG AGG AGA CGA TGA |
|  |  | overlap | GGCCCAGGCGGCC CTG ACT CAG CCG TCC TCG | GGC CGG CCT GGC CAC TAG TGG AGG AGA CGA TGA |
| **L39A** | **S** | fragment 1 | GGCCCAGGCGGCC CTG ACT CAG CCG TCC TCG | CTT CTG CTG ATA CCA GCC GTA |
|  |  | fragment 2 | TAC GGC TGG TAT CAG CAG AAG TGT CCT GGC AGT GCC CCT GTC | GGC CGG CCT GGC CAC TAG TGG AGG AGA CGA TGA |
|  |  | overlap | GGCCCAGGCGGCC CTG ACT CAG CCG TCC TCG | GGC CGG CCT GGC CAC TAG TGG AGG AGA CGA TGA |
| **L40** | **P** | fragment 1 | GGCCCAGGCGGCC CTG ACT CAG CCG TCC TCG | AGA CTT CTG CTG ATA CCA GCC |
|  |  | fragment 2 | GGC TGG TAT CAG CAG AAG TCT TGT GGC AGT GCC CCT GTC ACT | GGC CGG CCT GGC CAC TAG TGG AGG AGA CGA TGA |
|  |  | overlap | GGCCCAGGCGGCC CTG ACT CAG CCG TCC TCG | GGC CGG CCT GGC CAC TAG TGG AGG AGA CGA TGA |
| **L41** | **G** | fragment 1 | GGCCCAGGCGGCC CTG ACT CAG CCG TCC TCG | AGG AGA CTT CTG CTG ATA CCA |
|  |  | fragment 2 | TGG TAT CAG CAG AAG TCT CCT TGT AGT GCC CCT GTC ACT GTG | GGC CGG CCT GGC CAC TAG TGG AGG AGA CGA TGA |
|  |  | overlap | GGCCCAGGCGGCC CTG ACT CAG CCG TCC TCG | GGC CGG CCT GGC CAC TAG TGG AGG AGA CGA TGA |
| **L42** | **S** | fragment 1 | GGCCCAGGCGGCC CTG ACT CAG CCG TCC TCG | GCC AGG AGA CTT CTG CTG ATA |
|  |  | fragment 2 | TAT CAG CAG AAG TCT CCT GGC TGT GCC CCT GTC ACT GTG ATC | GGC CGG CCT GGC CAC TAG TGG AGG AGA CGA TGA |
|  |  | overlap | GGCCCAGGCGGCC CTG ACT CAG CCG TCC TCG | GGC CGG CCT GGC CAC TAG TGG AGG AGA CGA TGA |
| **L43** | **A** | fragment 1 | GGCCCAGGCGGCC CTG ACT CAG CCG TCC TCG | ACT GCC AGG AGA CTT CTG CTG |
|  |  | fragment 2 | CAG CAG AAG TCT CCT GGC AGT TGT CCT GTC ACT GTG ATC TAT | GGC CGG CCT GGC CAC TAG TGG AGG AGA CGA TGA |
|  |  | overlap | GGCCCAGGCGGCC CTG ACT CAG CCG TCC TCG | GGC CGG CCT GGC CAC TAG TGG AGG AGA CGA TGA |
| **L44** | **P** | fragment 1 | GGCCCAGGCGGCC CTG ACT CAG CCG TCC TCG | GGC ACT GCC AGG AGA CTT CTG |
|  |  | fragment 2 | CAG AAG TCT CCT GGC AGT GCC TGT GTC ACT GTG ATC TAT GAA | GGC CGG CCT GGC CAC TAG TGG AGG AGA CGA TGA |
|  |  | overlap | GGCCCAGGCGGCC CTG ACT CAG CCG TCC TCG | GGC CGG CCT GGC CAC TAG TGG AGG AGA CGA TGA |
| **L45** | **V** | fragment 1 | GGCCCAGGCGGCC CTG ACT CAG CCG TCC TCG | AGG GGC ACT GCC AGG AGA CTT |
|  |  | fragment 2 | AAG TCT CCT GGC AGT GCC CCT TGT ACT GTG ATC TAT GAA AGC | GGC CGG CCT GGC CAC TAG TGG AGG AGA CGA TGA |
|  |  | overlap | GGCCCAGGCGGCC CTG ACT CAG CCG TCC TCG | GGC CGG CCT GGC CAC TAG TGG AGG AGA CGA TGA |
| **L46** | **T** | fragment 1 | GGCCCAGGCGGCC CTG ACT CAG CCG TCC TCG | GAC AGG GGC ACT GCC AGG AGA |
|  |  | fragment 2 | TCT CCT GGC AGT GCC CCT GTC TGT GTG ATC TAT GAA AGC ACC | GGC CGG CCT GGC CAC TAG TGG AGG AGA CGA TGA |
|  |  | overlap | GGCCCAGGCGGCC CTG ACT CAG CCG TCC TCG | GGC CGG CCT GGC CAC TAG TGG AGG AGA CGA TGA |
| **L47** | **V** | fragment 1 | GGCCCAGGCGGCC CTG ACT CAG CCG TCC TCG | AGT GAC AGG GGC ACT GCC AGG |
|  |  | fragment 2 | CCT GGC AGT GCC CCT GTC ACT TGT ATC TAT GAA AGC ACC AAG | GGC CGG CCT GGC CAC TAG TGG AGG AGA CGA TGA |
|  |  | overlap | GGCCCAGGCGGCC CTG ACT CAG CCG TCC TCG | GGC CGG CCT GGC CAC TAG TGG AGG AGA CGA TGA |
| **L48** | **I** | fragment 1 | GGCCCAGGCGGCC CTG ACT CAG CCG TCC TCG | CAC AGT GAC AGG GGC ACT GCC |
|  |  | fragment 2 | GGC AGT GCC CCT GTC ACT GTG TGT TAT GAA AGC ACC AAG AGG | GGC CGG CCT GGC CAC TAG TGG AGG AGA CGA TGA |
|  |  | overlap | GGCCCAGGCGGCC CTG ACT CAG CCG TCC TCG | GGC CGG CCT GGC CAC TAG TGG AGG AGA CGA TGA |
| **L49** | **Y** | fragment 1 | GGCCCAGGCGGCC CTG ACT CAG CCG TCC TCG | GAT CAC AGT GAC AGG GGC ACT |
|  |  | fragment 2 | AGT GCC CCT GTC ACT GTG ATC TGT GAA AGC ACC AAG AGG CCC | GGC CGG CCT GGC CAC TAG TGG AGG AGA CGA TGA |
|  |  | overlap | GGCCCAGGCGGCC CTG ACT CAG CCG TCC TCG | GGC CGG CCT GGC CAC TAG TGG AGG AGA CGA TGA |
|  |  |  |  |  |
| **L57** | **N** | fragment 1 | GGCCCAGGCGGCC CTG ACT CAG CCG TCC TCG | CGA GGG CCT CTT GGT GCT TTC |
|  |  | fragment 2 | GAA AGC ACC AAG AGG CCC TCG TGT ATC CCT TCA CGA TTC TCC | GGC CGG CCT GGC CAC TAG TGG AGG AGA CGA TGA |
|  |  | overlap | GGCCCAGGCGGCC CTG ACT CAG CCG TCC TCG | GGC CGG CCT GGC CAC TAG TGG AGG AGA CGA TGA |
| **L58** | **I** | fragment 1 | GGCCCAGGCGGCC CTG ACT CAG CCG TCC TCG | GTT CGA GGG CCT CTT GGT GCT |
|  |  | fragment 2 | AGC ACC AAG AGG CCC TCG AAC TGT CCT TCA CGA TTC TCC GGT | GGC CGG CCT GGC CAC TAG TGG AGG AGA CGA TGA |
|  |  | overlap | GGCCCAGGCGGCC CTG ACT CAG CCG TCC TCG | GGC CGG CCT GGC CAC TAG TGG AGG AGA CGA TGA |
| **L59** | **P** | fragment 1 | GGCCCAGGCGGCC CTG ACT CAG CCG TCC TCG | GAT GTT CGA GGG CCT CTT GGT |
|  |  | fragment 2 | ACC AAG AGG CCC TCG AAC ATC TGT TCA CGA TTC TCC GGT TCC | GGC CGG CCT GGC CAC TAG TGG AGG AGA CGA TGA |
|  |  | overlap | GGCCCAGGCGGCC CTG ACT CAG CCG TCC TCG | GGC CGG CCT GGC CAC TAG TGG AGG AGA CGA TGA |
| **L60** | **S** | fragment 1 | GGCCCAGGCGGCC CTG ACT CAG CCG TCC TCG | AGG GAT GTT CGA GGG CCT CTT |
|  |  | fragment 2 | AAG AGG CCC TCG AAC ATC CCT TGT CGA TTC TCC GGT TCC ACA | GGC CGG CCT GGC CAC TAG TGG AGG AGA CGA TGA |
|  |  | overlap | GGCCCAGGCGGCC CTG ACT CAG CCG TCC TCG | GGC CGG CCT GGC CAC TAG TGG AGG AGA CGA TGA |
| **L61** | **R** | fragment 1 | GGCCCAGGCGGCC CTG ACT CAG CCG TCC TCG | TGA AGG GAT GTT CGA GGG CCT |
|  |  | fragment 2 | AGG CCC TCG AAC ATC CCT TCA TGT TTC TCC GGT TCC ACA TCT | GGC CGG CCT GGC CAC TAG TGG AGG AGA CGA TGA |
|  |  | overlap | GGCCCAGGCGGCC CTG ACT CAG CCG TCC TCG | GGC CGG CCT GGC CAC TAG TGG AGG AGA CGA TGA |
| **L62** | **F** | fragment 1 | GGCCCAGGCGGCC CTG ACT CAG CCG TCC TCG | TCG TGA AGG GAT GTT CGA GGG |
|  |  | fragment 2 | CCC TCG AAC ATC CCT TCA CGA TGT TCC GGT TCC ACA TCT GGC | GGC CGG CCT GGC CAC TAG TGG AGG AGA CGA TGA |
|  |  | overlap | GGCCCAGGCGGCC CTG ACT CAG CCG TCC TCG | GGC CGG CCT GGC CAC TAG TGG AGG AGA CGA TGA |
| **L63** | **S** | fragment 1 | GGCCCAGGCGGCC CTG ACT CAG CCG TCC TCG | GAA TCG TGA AGG GAT GTT CGA |
|  |  | fragment 2 | TCG AAC ATC CCT TCA CGA TTC TGT GGT TCC ACA TCT GGC TCC | GGC CGG CCT GGC CAC TAG TGG AGG AGA CGA TGA |
|  |  | overlap | GGCCCAGGCGGCC CTG ACT CAG CCG TCC TCG | GGC CGG CCT GGC CAC TAG TGG AGG AGA CGA TGA |
| **L64** | **G** | fragment 1 | GGCCCAGGCGGCC CTG ACT CAG CCG TCC TCG | GGA GAA TCG TGA AGG GAT GTT |
|  |  | fragment 2 | AAC ATC CCT TCA CGA TTC TCC TGT TCC ACA TCT GGC TCC ACG | GGC CGG CCT GGC CAC TAG TGG AGG AGA CGA TGA |
|  |  | overlap | GGCCCAGGCGGCC CTG ACT CAG CCG TCC TCG | GGC CGG CCT GGC CAC TAG TGG AGG AGA CGA TGA |
| **L65** | **S** | fragment 1 | GGCCCAGGCGGCC CTG ACT CAG CCG TCC TCG | ACC GGA GAA TCG TGA AGG GAT |
|  |  | fragment 2 | ATC CCT TCA CGA TTC TCC GGT TGT ACA TCT GGC TCC ACG GGC | GGC CGG CCT GGC CAC TAG TGG AGG AGA CGA TGA |
|  |  | overlap | GGCCCAGGCGGCC CTG ACT CAG CCG TCC TCG | GGC CGG CCT GGC CAC TAG TGG AGG AGA CGA TGA |
| **L66** | **T** | fragment 1 | GGCCCAGGCGGCC CTG ACT CAG CCG TCC TCG | GGA ACC GGA GAA TCG TGA AGG |
|  |  | fragment 2 | CCT TCA CGA TTC TCC GGT TCC TGT TCT GGC TCC ACG GGC ACA | GGC CGG CCT GGC CAC TAG TGG AGG AGA CGA TGA |
|  |  | overlap | GGCCCAGGCGGCC CTG ACT CAG CCG TCC TCG | GGC CGG CCT GGC CAC TAG TGG AGG AGA CGA TGA |
| **L67** | **S** | fragment 1 | GGCCCAGGCGGCC CTG ACT CAG CCG TCC TCG | TGT GGA ACC GGA GAA TCG TGA |
|  |  | fragment 2 | TCA CGA TTC TCC GGT TCC ACA TGT GGC TCC ACG GGC ACA TTA | GGC CGG CCT GGC CAC TAG TGG AGG AGA CGA TGA |
|  |  | overlap | GGCCCAGGCGGCC CTG ACT CAG CCG TCC TCG | GGC CGG CCT GGC CAC TAG TGG AGG AGA CGA TGA |
| **L68** | **G** | fragment 1 | GGCCCAGGCGGCC CTG ACT CAG CCG TCC TCG | AGA TGT GGA ACC GGA GAA TCG |
|  |  | fragment 2 | CGA TTC TCC GGT TCC ACA TCT TGT TCC ACG GGC ACA TTA ACC | GGC CGG CCT GGC CAC TAG TGG AGG AGA CGA TGA |
|  |  | overlap | GGCCCAGGCGGCC CTG ACT CAG CCG TCC TCG | GGC CGG CCT GGC CAC TAG TGG AGG AGA CGA TGA |
| **L69** | **S** | fragment 1 | GGCCCAGGCGGCC CTG ACT CAG CCG TCC TCG | GCC AGA TGT GGA ACC GGA GAA |
|  |  | fragment 2 | TTC TCC GGT TCC ACA TCT GGC TGT ACG GGC ACA TTA ACC ATC | GGC CGG CCT GGC CAC TAG TGG AGG AGA CGA TGA |
|  |  | overlap | GGCCCAGGCGGCC CTG ACT CAG CCG TCC TCG | GGC CGG CCT GGC CAC TAG TGG AGG AGA CGA TGA |
| **L70** | **T** | fragment 1 | GGCCCAGGCGGCC CTG ACT CAG CCG TCC TCG | GGA GCC AGA TGT GGA ACC GGA |
|  |  | fragment 2 | TCC GGT TCC ACA TCT GGC TCC TGT GGC ACA TTA ACC ATC ACT | GGC CGG CCT GGC CAC TAG TGG AGG AGA CGA TGA |
|  |  | overlap | GGCCCAGGCGGCC CTG ACT CAG CCG TCC TCG | GGC CGG CCT GGC CAC TAG TGG AGG AGA CGA TGA |
| **L71** | **G** | fragment 1 | GGCCCAGGCGGCC CTG ACT CAG CCG TCC TCG | CGT GGA GCC AGA TGT GGA ACC |
|  |  | fragment 2 | GGT TCC ACA TCT GGC TCC ACG TGT ACA TTA ACC ATC ACT GGG | GGC CGG CCT GGC CAC TAG TGG AGG AGA CGA TGA |
|  |  | overlap | GGCCCAGGCGGCC CTG ACT CAG CCG TCC TCG | GGC CGG CCT GGC CAC TAG TGG AGG AGA CGA TGA |
| **L72** | **T** | fragment 1 | GGCCCAGGCGGCC CTG ACT CAG CCG TCC TCG | GCC CGT GGA GCC AGA TGT GGA |
|  |  | fragment 2 | TCC ACA TCT GGC TCC ACG GGC TGT TTA ACC ATC ACT GGG GTC | GGC CGG CCT GGC CAC TAG TGG AGG AGA CGA TGA |
|  |  | overlap | GGCCCAGGCGGCC CTG ACT CAG CCG TCC TCG | GGC CGG CCT GGC CAC TAG TGG AGG AGA CGA TGA |
| **L73** | **L** | fragment 1 | GGCCCAGGCGGCC CTG ACT CAG CCG TCC TCG | TGT GCC CGT GGA GCC AGA TGT |
|  |  | fragment 2 | ACA TCT GGC TCC ACG GGC ACA TGT ACC ATC ACT GGG GTC CAA | GGC CGG CCT GGC CAC TAG TGG AGG AGA CGA TGA |
|  |  | overlap | GGCCCAGGCGGCC CTG ACT CAG CCG TCC TCG | GGC CGG CCT GGC CAC TAG TGG AGG AGA CGA TGA |
| **L74** | **T** | fragment 1 | GGCCCAGGCGGCC CTG ACT CAG CCG TCC TCG | TAA TGT GCC CGT GGA GCC AGA |
|  |  | fragment 2 | TCT GGC TCC ACG GGC ACA TTA TGT ATC ACT GGG GTC CAA GCC | GGC CGG CCT GGC CAC TAG TGG AGG AGA CGA TGA |
|  |  | overlap | GGCCCAGGCGGCC CTG ACT CAG CCG TCC TCG | GGC CGG CCT GGC CAC TAG TGG AGG AGA CGA TGA |
| **L75** | **I** | fragment 1 | GGCCCAGGCGGCC CTG ACT CAG CCG TCC TCG | GGT TAA TGT GCC CGT GGA GCC |
|  |  | fragment 2 | GGC TCC ACG GGC ACA TTA ACC TGT ACT GGG GTC CAA GCC GAT | GGC CGG CCT GGC CAC TAG TGG AGG AGA CGA TGA |
|  |  | overlap | GGCCCAGGCGGCC CTG ACT CAG CCG TCC TCG | GGC CGG CCT GGC CAC TAG TGG AGG AGA CGA TGA |
| **L76** | **T** | fragment 1 | GGCCCAGGCGGCC CTG ACT CAG CCG TCC TCG | GAT GGT TAA TGT GCC CGT GGA |
|  |  | fragment 2 | TCC ACG GGC ACA TTA ACC ATC TGT GGG GTC CAA GCC GAT GAC | GGC CGG CCT GGC CAC TAG TGG AGG AGA CGA TGA |
|  |  | overlap | GGCCCAGGCGGCC CTG ACT CAG CCG TCC TCG | GGC CGG CCT GGC CAC TAG TGG AGG AGA CGA TGA |
| **L77** | **G** | fragment 1 | GGCCCAGGCGGCC CTG ACT CAG CCG TCC TCG | AGT GAT GGT TAA TGT GCC CGT |
|  |  | fragment 2 | ACG GGC ACA TTA ACC ATC ACT TGT GTC CAA GCC GAT GAC GCG | GGC CGG CCT GGC CAC TAG TGG AGG AGA CGA TGA |
|  |  | overlap | GGCCCAGGCGGCC CTG ACT CAG CCG TCC TCG | GGC CGG CCT GGC CAC TAG TGG AGG AGA CGA TGA |
| **L78** | **V** | fragment 1 | GGCCCAGGCGGCC CTG ACT CAG CCG TCC TCG | CCC AGT GAT GGT TAA TGT GCC |
|  |  | fragment 2 | GGC ACA TTA ACC ATC ACT GGG TGT CAA GCC GAT GAC GCG GCT | GGC CGG CCT GGC CAC TAG TGG AGG AGA CGA TGA |
|  |  | overlap | GGCCCAGGCGGCC CTG ACT CAG CCG TCC TCG | GGC CGG CCT GGC CAC TAG TGG AGG AGA CGA TGA |
| **L79** | **Q** | fragment 1 | GGCCCAGGCGGCC CTG ACT CAG CCG TCC TCG | GAC CCC AGT GAT GGT TAA TGT |
|  |  | fragment 2 | ACA TTA ACC ATC ACT GGG GTC TGT GCC GAT GAC GCG GCT GTC | GGC CGG CCT GGC CAC TAG TGG AGG AGA CGA TGA |
|  |  | overlap | GGCCCAGGCGGCC CTG ACT CAG CCG TCC TCG | GGC CGG CCT GGC CAC TAG TGG AGG AGA CGA TGA |
| **L80** | **A** | fragment 1 | GGCCCAGGCGGCC CTG ACT CAG CCG TCC TCG | TTG GAC CCC AGT GAT GGT TAA |
|  |  | fragment 2 | TTA ACC ATC ACT GGG GTC CAA TGT GAT GAC GCG GCT GTC TAT | GGC CGG CCT GGC CAC TAG TGG AGG AGA CGA TGA |
|  |  | overlap | GGCCCAGGCGGCC CTG ACT CAG CCG TCC TCG | GGC CGG CCT GGC CAC TAG TGG AGG AGA CGA TGA |
| **L81** | **D** | fragment 1 | GGCCCAGGCGGCC CTG ACT CAG CCG TCC TCG | GGC TTG GAC CCC AGT GAT GGT |
|  |  | fragment 2 | ACC ATC ACT GGG GTC CAA GCC TGT GAC GCG GCT GTC TAT TAT | GGC CGG CCT GGC CAC TAG TGG AGG AGA CGA TGA |
|  |  | overlap | GGCCCAGGCGGCC CTG ACT CAG CCG TCC TCG | GGC CGG CCT GGC CAC TAG TGG AGG AGA CGA TGA |
| **L82** | **D** | fragment 1 | GGCCCAGGCGGCC CTG ACT CAG CCG TCC TCG | ATC GGC TTG GAC CCC AGT GAT |
|  |  | fragment 2 | ATC ACT GGG GTC CAA GCC GAT TGT GCG GCT GTC TAT TAT TGT | GGC CGG CCT GGC CAC TAG TGG AGG AGA CGA TGA |
|  |  | overlap | GGCCCAGGCGGCC CTG ACT CAG CCG TCC TCG | GGC CGG CCT GGC CAC TAG TGG AGG AGA CGA TGA |
| **L83** | **A** | fragment 1 | GGCCCAGGCGGCC CTG ACT CAG CCG TCC TCG | GTC ATC GGC TTG GAC CCC AGT |
|  |  | fragment 2 | ACT GGG GTC CAA GCC GAT GAC TGT GCT GTC TAT TAT TGT GGG | GGC CGG CCT GGC CAC TAG TGG AGG AGA CGA TGA |
|  |  | overlap | GGCCCAGGCGGCC CTG ACT CAG CCG TCC TCG | GGC CGG CCT GGC CAC TAG TGG AGG AGA CGA TGA |
| **L84** | **A** | fragment 1 | GGCCCAGGCGGCC CTG ACT CAG CCG TCC TCG | CGC GTC ATC GGC TTG GAC CCC |
|  |  | fragment 2 | GGG GTC CAA GCC GAT GAC GCG TGT GTC TAT TAT TGT GGG AGC | GGC CGG CCT GGC CAC TAG TGG AGG AGA CGA TGA |
|  |  | overlap | GGCCCAGGCGGCC CTG ACT CAG CCG TCC TCG | GGC CGG CCT GGC CAC TAG TGG AGG AGA CGA TGA |
| **L85** | **V** | fragment 1 | GGCCCAGGCGGCC CTG ACT CAG CCG TCC TCG | AGC CGC GTC ATC GGC TTG GAC |
|  |  | fragment 2 | GTC CAA GCC GAT GAC GCG GCT TGT TAT TAT TGT GGG AGC TGG | GGC CGG CCT GGC CAC TAG TGG AGG AGA CGA TGA |
|  |  | overlap | GGCCCAGGCGGCC CTG ACT CAG CCG TCC TCG | GGC CGG CCT GGC CAC TAG TGG AGG AGA CGA TGA |
| **L86** | **Y** | fragment 1 | GGCCCAGGCGGCC CTG ACT CAG CCG TCC TCG | GAC AGC CGC GTC ATC GGC TTG |
|  |  | fragment 2 | CAA GCC GAT GAC GCG GCT GTC TGT TAT TGT GGG AGC TGG GAC | GGC CGG CCT GGC CAC TAG TGG AGG AGA CGA TGA |
|  |  | overlap | GGCCCAGGCGGCC CTG ACT CAG CCG TCC TCG | GGC CGG CCT GGC CAC TAG TGG AGG AGA CGA TGA |
| **L87** | **Y** | fragment 1 | GGCCCAGGCGGCC CTG ACT CAG CCG TCC TCG | ATA GAC AGC CGC GTC ATC GGC |
|  |  | fragment 2 | GCC GAT GAC GCG GCT GTC TAT TGT TGT GGG AGC TGG GAC AGC | GGC CGG CCT GGC CAC TAG TGG AGG AGA CGA TGA |
|  |  | overlap | GGCCCAGGCGGCC CTG ACT CAG CCG TCC TCG | GGC CGG CCT GGC CAC TAG TGG AGG AGA CGA TGA |
|  |  |  |  |  |
| **L98** | **F** | fragment 1 | GGCCCAGGCGGCC CTG ACT CAG CCG TCC TCG | TAT ACC AAC ACT ACT GCT GTC |
|  |  | fragment 2 | GAC AGC AGT AGT GTT GGT ATA TGT GGG GCC GGG ACA ACC CTG | GGC CGG CCT GGC CAC TAG TGG AGG AGA CGA TGA |
|  |  | overlap | GGCCCAGGCGGCC CTG ACT CAG CCG TCC TCG | GGC CGG CCT GGC CAC TAG TGG AGG AGA CGA TGA |
| **L99** | **G** | fragment 1 | GGCCCAGGCGGCC CTG ACT CAG CCG TCC TCG | AAA TAT ACC AAC ACT ACT GCT |
|  |  | fragment 2 | AGC AGT AGT GTT GGT ATA TTT TGT GCC GGG ACA ACC CTG ACC | GGC CGG CCT GGC CAC TAG TGG AGG AGA CGA TGA |
|  |  | overlap | GGCCCAGGCGGCC CTG ACT CAG CCG TCC TCG | GGC CGG CCT GGC CAC TAG TGG AGG AGA CGA TGA |
| **L100** | **A** | fragment 1 | GGCCCAGGCGGCC CTG ACT CAG CCG TCC TCG | CCC AAA TAT ACC AAC ACT ACT |
|  |  | fragment 2 | AGT AGT GTT GGT ATA TTT GGG TGT GGG ACA ACC CTG ACC GTC | GGC CGG CCT GGC CAC TAG TGG AGG AGA CGA TGA |
|  |  | overlap | GGCCCAGGCGGCC CTG ACT CAG CCG TCC TCG | GGC CGG CCT GGC CAC TAG TGG AGG AGA CGA TGA |
| **L101** | **G** | fragment 1 | GGCCCAGGCGGCC CTG ACT CAG CCG TCC TCG | GGC CCC AAA TAT ACC AAC ACT |
|  |  | fragment 2 | AGT GTT GGT ATA TTT GGG GCC TGT ACA ACC CTG ACC GTC CTA | GGC CGG CCT GGC CAC TAG TGG AGG AGA CGA TGA |
|  |  | overlap | GGCCCAGGCGGCC CTG ACT CAG CCG TCC TCG | GGC CGG CCT GGC CAC TAG TGG AGG AGA CGA TGA |
| **L102** | **T** | fragment 1 | GGCCCAGGCGGCC CTG ACT CAG CCG TCC TCG | CCC GGC CCC AAA TAT ACC AAC |
|  |  | fragment 2 | GTT GGT ATA TTT GGG GCC GGG TGT ACC CTG ACC GTC CTA GGT | GGC CGG CCT GGC CAC TAG TGG AGG AGA CGA TGA |
|  |  | overlap | GGCCCAGGCGGCC CTG ACT CAG CCG TCC TCG | GGC CGG CCT GGC CAC TAG TGG AGG AGA CGA TGA |
| **L103** | **T** | fragment 1 | GGCCCAGGCGGCC CTG ACT CAG CCG TCC TCG | TGT CCC GGC CCC AAA TAT ACC |
|  |  | fragment 2 | GGT ATA TTT GGG GCC GGG ACA TGT CTG ACC GTC CTA GGT CAG | GGC CGG CCT GGC CAC TAG TGG AGG AGA CGA TGA |
|  |  | overlap | GGCCCAGGCGGCC CTG ACT CAG CCG TCC TCG | GGC CGG CCT GGC CAC TAG TGG AGG AGA CGA TGA |
| **L104** | **L** | fragment 1 | GGCCCAGGCGGCC CTG ACT CAG CCG TCC TCG | GGT TGT CCC GGC CCC AAA TAT |
|  |  | fragment 2 | ATA TTT GGG GCC GGG ACA ACC TGT ACC GTC CTA GGT CAG TCC | GGC CGG CCT GGC CAC TAG TGG AGG AGA CGA TGA |
|  |  | overlap | GGCCCAGGCGGCC CTG ACT CAG CCG TCC TCG | GGC CGG CCT GGC CAC TAG TGG AGG AGA CGA TGA |
| **L105** | **T** | fragment 1 | GGCCCAGGCGGCC CTG ACT CAG CCG TCC TCG | CAG GGT TGT CCC GGC CCC AAA |
|  |  | fragment 2 | TTT GGG GCC GGG ACA ACC CTG TGT GTC CTA GGT CAG TCC TCT | GGC CGG CCT GGC CAC TAG TGG AGG AGA CGA TGA |
|  |  | overlap | GGCCCAGGCGGCC CTG ACT CAG CCG TCC TCG | GGC CGG CCT GGC CAC TAG TGG AGG AGA CGA TGA |
| **L106** | **V** | fragment 1 | GGCCCAGGCGGCC CTG ACT CAG CCG TCC TCG | GGT CAG GGT TGT CCC GGC CCC |
|  |  | fragment 2 | GGG GCC GGG ACA ACC CTG ACC TGT CTA GGT CAG TCC TCT AGA | GGC CGG CCT GGC CAC TAG TGG AGG AGA CGA TGA |
|  |  | overlap | GGCCCAGGCGGCC CTG ACT CAG CCG TCC TCG | GGC CGG CCT GGC CAC TAG TGG AGG AGA CGA TGA |
| **L106A** | **L** | fragment 1 | GGCCCAGGCGGCC CTG ACT CAG CCG TCC TCG | GAC GGT CAG GGT TGT CCC GGC |
|  |  | fragment 2 | GCC GGG ACA ACC CTG ACC GTC TGT GGT CAG TCC TCT AGA TCT | GGC CGG CCT GGC CAC TAG TGG AGG AGA CGA TGA |
|  |  | overlap | GGCCCAGGCGGCC CTG ACT CAG CCG TCC TCG | GGC CGG CCT GGC CAC TAG TGG AGG AGA CGA TGA |
|  |  |  |  |  |
| **H1** | **A** | fragment 1 | GGCCCAGGCGGCC CTG ACT CAG CCG TCC TCG | GGA ACC GCC ACC ACC GGA GCT |
|  |  | fragment 2 | AGC TCC GGT GGT GGC GGT TCC TGT GTG ACG TTG GAC GAG TCC | GGC CGG CCT GGC CAC TAG TGG AGG AGA CGA TGA |
|  |  | overlap | GGCCCAGGCGGCC CTG ACT CAG CCG TCC TCG | GGC CGG CCT GGC CAC TAG TGG AGG AGA CGA TGA |
| **H2** | **V** | fragment 1 | GGCCCAGGCGGCC CTG ACT CAG CCG TCC TCG | GGC GGA ACC GCC ACC ACC GGA |
|  |  | fragment 2 | TCC GGT GGT GGC GGT TCC GCC TGT ACG TTG GAC GAG TCC GGG | GGC CGG CCT GGC CAC TAG TGG AGG AGA CGA TGA |
|  |  | overlap | GGCCCAGGCGGCC CTG ACT CAG CCG TCC TCG | GGC CGG CCT GGC CAC TAG TGG AGG AGA CGA TGA |
| **H3** | **T** | fragment 1 | GGCCCAGGCGGCC CTG ACT CAG CCG TCC TCG | CAC GGC GGA ACC GCC ACC ACC |
|  |  | fragment 2 | GGT GGT GGC GGT TCC GCC GTG TGT TTG GAC GAG TCC GGG GGC | GGC CGG CCT GGC CAC TAG TGG AGG AGA CGA TGA |
|  |  | overlap | GGCCCAGGCGGCC CTG ACT CAG CCG TCC TCG | GGC CGG CCT GGC CAC TAG TGG AGG AGA CGA TGA |
| **H4** | **L** | fragment 1 | GGCCCAGGCGGCC CTG ACT CAG CCG TCC TCG | CGT CAC GGC GGA ACC GCC ACC |
|  |  | fragment 2 | GGT GGC GGT TCC GCC GTG ACG TGT GAC GAG TCC GGG GGC GGC | GGC CGG CCT GGC CAC TAG TGG AGG AGA CGA TGA |
|  |  | overlap | GGCCCAGGCGGCC CTG ACT CAG CCG TCC TCG | GGC CGG CCT GGC CAC TAG TGG AGG AGA CGA TGA |
| **H5** | **D** | fragment 1 | GGCCCAGGCGGCC CTG ACT CAG CCG TCC TCG | CAA CGT CAC GGC GGA ACC GCC |
|  |  | fragment 2 | GGC GGT TCC GCC GTG ACG TTG TGT GAG TCC GGG GGC GGC CTC | GGC CGG CCT GGC CAC TAG TGG AGG AGA CGA TGA |
|  |  | overlap | GGCCCAGGCGGCC CTG ACT CAG CCG TCC TCG | GGC CGG CCT GGC CAC TAG TGG AGG AGA CGA TGA |
| **H6** | **E** | fragment 1 | GGCCCAGGCGGCC CTG ACT CAG CCG TCC TCG | GTC CAA CGT CAC GGC GGA ACC |
|  |  | fragment 2 | GGT TCC GCC GTG ACG TTG GAC TGT TCC GGG GGC GGC CTC CAG | GGC CGG CCT GGC CAC TAG TGG AGG AGA CGA TGA |
|  |  | overlap | GGCCCAGGCGGCC CTG ACT CAG CCG TCC TCG | GGC CGG CCT GGC CAC TAG TGG AGG AGA CGA TGA |
| **H7** | **S** | fragment 1 | GGCCCAGGCGGCC CTG ACT CAG CCG TCC TCG | CTC GTC CAA CGT CAC GGC GGA |
|  |  | fragment 2 | TCC GCC GTG ACG TTG GAC GAG TGT GGG GGC GGC CTC CAG ACG | GGC CGG CCT GGC CAC TAG TGG AGG AGA CGA TGA |
|  |  | overlap | GGCCCAGGCGGCC CTG ACT CAG CCG TCC TCG | GGC CGG CCT GGC CAC TAG TGG AGG AGA CGA TGA |
| **H8** | **G** | fragment 1 | GGCCCAGGCGGCC CTG ACT CAG CCG TCC TCG | GGA CTC GTC CAA CGT CAC GGC |
|  |  | fragment 2 | GCC GTG ACG TTG GAC GAG TCC TGT GGC GGC CTC CAG ACG CCC | GGC CGG CCT GGC CAC TAG TGG AGG AGA CGA TGA |
|  |  | overlap | GGCCCAGGCGGCC CTG ACT CAG CCG TCC TCG | GGC CGG CCT GGC CAC TAG TGG AGG AGA CGA TGA |
| **H9** | **G** | fragment 1 | GGCCCAGGCGGCC CTG ACT CAG CCG TCC TCG | CCC GGA CTC GTC CAA CGT CAC |
|  |  | fragment 2 | GTG ACG TTG GAC GAG TCC GGG TGT GGC CTC CAG ACG CCC GGA | GGC CGG CCT GGC CAC TAG TGG AGG AGA CGA TGA |
|  |  | overlap | GGCCCAGGCGGCC CTG ACT CAG CCG TCC TCG | GGC CGG CCT GGC CAC TAG TGG AGG AGA CGA TGA |
| **H10** | **G** | fragment 1 | GGCCCAGGCGGCC CTG ACT CAG CCG TCC TCG | GCC CCC GGA CTC GTC CAA CGT |
|  |  | fragment 2 | ACG TTG GAC GAG TCC GGG GGC TGT CTC CAG ACG CCC GGA GGA | GGC CGG CCT GGC CAC TAG TGG AGG AGA CGA TGA |
|  |  | overlap | GGCCCAGGCGGCC CTG ACT CAG CCG TCC TCG | GGC CGG CCT GGC CAC TAG TGG AGG AGA CGA TGA |
| **H11** | **L** | fragment 1 | GGCCCAGGCGGCC CTG ACT CAG CCG TCC TCG | GCC GCC CCC GGA CTC GTC CAA |
|  |  | fragment 2 | TTG GAC GAG TCC GGG GGC GGC TGT CAG ACG CCC GGA GGA GCG | GGC CGG CCT GGC CAC TAG TGG AGG AGA CGA TGA |
|  |  | overlap | GGCCCAGGCGGCC CTG ACT CAG CCG TCC TCG | GGC CGG CCT GGC CAC TAG TGG AGG AGA CGA TGA |
| **H12** | **Q** | fragment 1 | GGCCCAGGCGGCC CTG ACT CAG CCG TCC TCG | GAG GCC GCC CCC GGA CTC GTC |
|  |  | fragment 2 | GAC GAG TCC GGG GGC GGC CTC TGT ACG CCC GGA GGA GCG CTC | GGC CGG CCT GGC CAC TAG TGG AGG AGA CGA TGA |
|  |  | overlap | GGCCCAGGCGGCC CTG ACT CAG CCG TCC TCG | GGC CGG CCT GGC CAC TAG TGG AGG AGA CGA TGA |
| **H13** | **T** | fragment 1 | GGCCCAGGCGGCC CTG ACT CAG CCG TCC TCG | CTG GAG GCC GCC CCC GGA CTC |
|  |  | fragment 2 | GAG TCC GGG GGC GGC CTC CAG TGT CCC GGA GGA GCG CTC AGC | GGC CGG CCT GGC CAC TAG TGG AGG AGA CGA TGA |
|  |  | overlap | GGCCCAGGCGGCC CTG ACT CAG CCG TCC TCG | GGC CGG CCT GGC CAC TAG TGG AGG AGA CGA TGA |
| **H14** | **P** | fragment 1 | GGCCCAGGCGGCC CTG ACT CAG CCG TCC TCG | CGT CTG GAG GCC GCC CCC GGA |
|  |  | fragment 2 | TCC GGG GGC GGC CTC CAG ACG TGT GGA GGA GCG CTC AGC CTC | GGC CGG CCT GGC CAC TAG TGG AGG AGA CGA TGA |
|  |  | overlap | GGCCCAGGCGGCC CTG ACT CAG CCG TCC TCG | GGC CGG CCT GGC CAC TAG TGG AGG AGA CGA TGA |
| **H15** | **G** | fragment 1 | GGCCCAGGCGGCC CTG ACT CAG CCG TCC TCG | GGG CGT CTG GAG GCC GCC CCC |
|  |  | fragment 2 | GGG GGC GGC CTC CAG ACG CCC TGT GGA GCG CTC AGC CTC GTC | GGC CGG CCT GGC CAC TAG TGG AGG AGA CGA TGA |
|  |  | overlap | GGCCCAGGCGGCC CTG ACT CAG CCG TCC TCG | GGC CGG CCT GGC CAC TAG TGG AGG AGA CGA TGA |
| **H16** | **G** | fragment 1 | GGCCCAGGCGGCC CTG ACT CAG CCG TCC TCG | TCC GGG CGT CTG GAG GCC GCC |
|  |  | fragment 2 | GGC GGC CTC CAG ACG CCC GGA TGT GCG CTC AGC CTC GTC TGC | GGC CGG CCT GGC CAC TAG TGG AGG AGA CGA TGA |
|  |  | overlap | GGCCCAGGCGGCC CTG ACT CAG CCG TCC TCG | GGC CGG CCT GGC CAC TAG TGG AGG AGA CGA TGA |
| **H17** | **A** | fragment 1 | GGCCCAGGCGGCC CTG ACT CAG CCG TCC TCG | TCC TCC GGG CGT CTG GAG GCC |
|  |  | fragment 2 | GGC CTC CAG ACG CCC GGA GGA TGT CTC AGC CTC GTC TGC AAG | GGC CGG CCT GGC CAC TAG TGG AGG AGA CGA TGA |
|  |  | overlap | GGCCCAGGCGGCC CTG ACT CAG CCG TCC TCG | GGC CGG CCT GGC CAC TAG TGG AGG AGA CGA TGA |
| **H18** | **L** | fragment 1 | GGCCCAGGCGGCC CTG ACT CAG CCG TCC TCG | CGC TCC TCC GGG CGT CTG GAG |
|  |  | fragment 2 | CTC CAG ACG CCC GGA GGA GCG TGT AGC CTC GTC TGC AAG GCC | GGC CGG CCT GGC CAC TAG TGG AGG AGA CGA TGA |
|  |  | overlap | GGCCCAGGCGGCC CTG ACT CAG CCG TCC TCG | GGC CGG CCT GGC CAC TAG TGG AGG AGA CGA TGA |
| **H19** | **S** | fragment 1 | GGCCCAGGCGGCC CTG ACT CAG CCG TCC TCG | GAG CGC TCC TCC GGG CGT CTG |
|  |  | fragment 2 | CAG ACG CCC GGA GGA GCG CTC TGT CTC GTC TGC AAG GCC TCC | GGC CGG CCT GGC CAC TAG TGG AGG AGA CGA TGA |
|  |  | overlap | GGCCCAGGCGGCC CTG ACT CAG CCG TCC TCG | GGC CGG CCT GGC CAC TAG TGG AGG AGA CGA TGA |
| **H20** | **L** | fragment 1 | GGCCCAGGCGGCC CTG ACT CAG CCG TCC TCG | GCT GAG CGC TCC TCC GGG CGT |
|  |  | fragment 2 | ACG CCC GGA GGA GCG CTC AGC TGT GTC TGC AAG GCC TCC GGG | GGC CGG CCT GGC CAC TAG TGG AGG AGA CGA TGA |
|  |  | overlap | GGCCCAGGCGGCC CTG ACT CAG CCG TCC TCG | GGC CGG CCT GGC CAC TAG TGG AGG AGA CGA TGA |
| **H21** | **V** | fragment 1 | GGCCCAGGCGGCC CTG ACT CAG CCG TCC TCG | GAG GCT GAG CGC TCC TCC GGG |
|  |  | fragment 2 | CCC GGA GGA GCG CTC AGC CTC TGT TGC AAG GCC TCC GGG TTC | GGC CGG CCT GGC CAC TAG TGG AGG AGA CGA TGA |
|  |  | overlap | GGCCCAGGCGGCC CTG ACT CAG CCG TCC TCG | GGC CGG CCT GGC CAC TAG TGG AGG AGA CGA TGA |
| **H23** | **K** | fragment 1 | GGCCCAGGCGGCC CTG ACT CAG CCG TCC TCG | GCA GAC GAG GCT GAG CGC TCC |
|  |  | fragment 2 | GGA GCG CTC AGC CTC GTC TGC TGT GCC TCC GGG TTC ACC TTC | GGC CGG CCT GGC CAC TAG TGG AGG AGA CGA TGA |
|  |  | overlap | GGCCCAGGCGGCC CTG ACT CAG CCG TCC TCG | GGC CGG CCT GGC CAC TAG TGG AGG AGA CGA TGA |
| **H24** | **A** | fragment 1 | GGCCCAGGCGGCC CTG ACT CAG CCG TCC TCG | CTT GCA GAC GAG GCT GAG CGC |
|  |  | fragment 2 | GCG CTC AGC CTC GTC TGC AAG TGT TCC GGG TTC ACC TTC AGC | GGC CGG CCT GGC CAC TAG TGG AGG AGA CGA TGA |
|  |  | overlap | GGCCCAGGCGGCC CTG ACT CAG CCG TCC TCG | GGC CGG CCT GGC CAC TAG TGG AGG AGA CGA TGA |
| **H25** | **S** | fragment 1 | GGCCCAGGCGGCC CTG ACT CAG CCG TCC TCG | GGC CTT GCA GAC GAG GCT GAG |
|  |  | fragment 2 | CTC AGC CTC GTC TGC AAG GCC TGT GGG TTC ACC TTC AGC AGT | GGC CGG CCT GGC CAC TAG TGG AGG AGA CGA TGA |
|  |  | overlap | GGCCCAGGCGGCC CTG ACT CAG CCG TCC TCG | GGC CGG CCT GGC CAC TAG TGG AGG AGA CGA TGA |
|  |  |  |  |  |
| **H36** | **W** | fragment 1 | GGCCCAGGCGGCC CTG ACT CAG CCG TCC TCG | TCC CAT GTC GTA ACT GCT GAA |
|  |  | fragment 2 | TTC AGC AGT TAC GAC ATG GGA TGT GTG CGA CAG GCG CCC GGC | GGC CGG CCT GGC CAC TAG TGG AGG AGA CGA TGA |
|  |  | overlap | GGCCCAGGCGGCC CTG ACT CAG CCG TCC TCG | GGC CGG CCT GGC CAC TAG TGG AGG AGA CGA TGA |
| **H37** | **V** | fragment 1 | GGCCCAGGCGGCC CTG ACT CAG CCG TCC TCG | CCA TCC CAT GTC GTA ACT GCT |
|  |  | fragment 2 | AGC AGT TAC GAC ATG GGA TGG TGT CGA CAG GCG CCC GGC AAA | GGC CGG CCT GGC CAC TAG TGG AGG AGA CGA TGA |
|  |  | overlap | GGCCCAGGCGGCC CTG ACT CAG CCG TCC TCG | GGC CGG CCT GGC CAC TAG TGG AGG AGA CGA TGA |
| **H38** | **R** | fragment 1 | GGCCCAGGCGGCC CTG ACT CAG CCG TCC TCG | CAC CCA TCC CAT GTC GTA ACT |
|  |  | fragment 2 | AGT TAC GAC ATG GGA TGG GTG TGT CAG GCG CCC GGC AAA GGG | GGC CGG CCT GGC CAC TAG TGG AGG AGA CGA TGA |
|  |  | overlap | GGCCCAGGCGGCC CTG ACT CAG CCG TCC TCG | GGC CGG CCT GGC CAC TAG TGG AGG AGA CGA TGA |
| **H39** | **Q** | fragment 1 | GGCCCAGGCGGCC CTG ACT CAG CCG TCC TCG | TCG CAC CCA TCC CAT GTC GTA |
|  |  | fragment 2 | TAC GAC ATG GGA TGG GTG CGA TGT GCG CCC GGC AAA GGG CTG | GGC CGG CCT GGC CAC TAG TGG AGG AGA CGA TGA |
|  |  | overlap | GGCCCAGGCGGCC CTG ACT CAG CCG TCC TCG | GGC CGG CCT GGC CAC TAG TGG AGG AGA CGA TGA |
| **H40** | **A** | fragment 1 | GGCCCAGGCGGCC CTG ACT CAG CCG TCC TCG | CTG TCG CAC CCA TCC CAT GTC |
|  |  | fragment 2 | GAC ATG GGA TGG GTG CGA CAG TGT CCC GGC AAA GGG CTG GAA | GGC CGG CCT GGC CAC TAG TGG AGG AGA CGA TGA |
|  |  | overlap | GGCCCAGGCGGCC CTG ACT CAG CCG TCC TCG | GGC CGG CCT GGC CAC TAG TGG AGG AGA CGA TGA |
| **H41** | **P** | fragment 1 | GGCCCAGGCGGCC CTG ACT CAG CCG TCC TCG | CGC CTG TCG CAC CCA TCC CAT |
|  |  | fragment 2 | ATG GGA TGG GTG CGA CAG GCG TGT GGC AAA GGG CTG GAA TGG | GGC CGG CCT GGC CAC TAG TGG AGG AGA CGA TGA |
|  |  | overlap | GGCCCAGGCGGCC CTG ACT CAG CCG TCC TCG | GGC CGG CCT GGC CAC TAG TGG AGG AGA CGA TGA |
| **H42** | **G** | fragment 1 | GGCCCAGGCGGCC CTG ACT CAG CCG TCC TCG | GGG CGC CTG TCG CAC CCA TCC |
|  |  | fragment 2 | GGA TGG GTG CGA CAG GCG CCC TGT AAA GGG CTG GAA TGG GTC | GGC CGG CCT GGC CAC TAG TGG AGG AGA CGA TGA |
|  |  | overlap | GGCCCAGGCGGCC CTG ACT CAG CCG TCC TCG | GGC CGG CCT GGC CAC TAG TGG AGG AGA CGA TGA |
| **H43** | **K** | fragment 1 | GGCCCAGGCGGCC CTG ACT CAG CCG TCC TCG | GCC GGG CGC CTG TCG CAC CCA |
|  |  | fragment 2 | TGG GTG CGA CAG GCG CCC GGC TGT GGG CTG GAA TGG GTC GCT | GGC CGG CCT GGC CAC TAG TGG AGG AGA CGA TGA |
|  |  | overlap | GGCCCAGGCGGCC CTG ACT CAG CCG TCC TCG | GGC CGG CCT GGC CAC TAG TGG AGG AGA CGA TGA |
| **H44** | **G** | fragment 1 | GGCCCAGGCGGCC CTG ACT CAG CCG TCC TCG | TTT GCC GGG CGC CTG TCG CAC |
|  |  | fragment 2 | GTG CGA CAG GCG CCC GGC AAA TGT CTG GAA TGG GTC GCT GGT | GGC CGG CCT GGC CAC TAG TGG AGG AGA CGA TGA |
|  |  | overlap | GGCCCAGGCGGCC CTG ACT CAG CCG TCC TCG | GGC CGG CCT GGC CAC TAG TGG AGG AGA CGA TGA |
| **H45** | **L** | fragment 1 | GGCCCAGGCGGCC CTG ACT CAG CCG TCC TCG | CCC TTT GCC GGG CGC CTG TCG |
|  |  | fragment 2 | CGA CAG GCG CCC GGC AAA GGG TGT GAA TGG GTC GCT GGT ATT | GGC CGG CCT GGC CAC TAG TGG AGG AGA CGA TGA |
|  |  | overlap | GGCCCAGGCGGCC CTG ACT CAG CCG TCC TCG | GGC CGG CCT GGC CAC TAG TGG AGG AGA CGA TGA |
| **H46** | **E** | fragment 1 | GGCCCAGGCGGCC CTG ACT CAG CCG TCC TCG | TTC CAG CCC TTT GCC GGG CGC |
|  |  | fragment 2 | GCG CCC GGC AAA GGG CTG GAA TGT GTC GCT GGT ATT GAT GAT | GGC CGG CCT GGC CAC TAG TGG AGG AGA CGA TGA |
|  |  | overlap | GGCCCAGGCGGCC CTG ACT CAG CCG TCC TCG | GGC CGG CCT GGC CAC TAG TGG AGG AGA CGA TGA |
| **H47** | **W** | fragment 1 | GGCCCAGGCGGCC CTG ACT CAG CCG TCC TCG | CCA TTC CAG CCC TTT GCC GGG |
|  |  | fragment 2 | CCC GGC AAA GGG CTG GAA TGG TGT GCT GGT ATT GAT GAT GAT | GGC CGG CCT GGC CAC TAG TGG AGG AGA CGA TGA |
|  |  | overlap | GGCCCAGGCGGCC CTG ACT CAG CCG TCC TCG | GGC CGG CCT GGC CAC TAG TGG AGG AGA CGA TGA |
| **H48** | **V** | fragment 1 | GGCCCAGGCGGCC CTG ACT CAG CCG TCC TCG | GAC CCA TTC CAG CCC TTT GCC |
|  |  | fragment 2 | GGC AAA GGG CTG GAA TGG GTC TGT GGT ATT GAT GAT GAT GGT | GGC CGG CCT GGC CAC TAG TGG AGG AGA CGA TGA |
|  |  | overlap | GGCCCAGGCGGCC CTG ACT CAG CCG TCC TCG | GGC CGG CCT GGC CAC TAG TGG AGG AGA CGA TGA |
| **H49** | **A** | fragment 1 | GGCCCAGGCGGCC CTG ACT CAG CCG TCC TCG | AGC GAC CCA TTC CAG CCC TTT |
|  |  | fragment 2 | AAA GGG CTG GAA TGG GTC GCT TGT ATT GAT GAT GAT GGT AGT | GGC CGG CCT GGC CAC TAG TGG AGG AGA CGA TGA |
|  |  | overlap | GGCCCAGGCGGCC CTG ACT CAG CCG TCC TCG | GGC CGG CCT GGC CAC TAG TGG AGG AGA CGA TGA |
|  |  |  |  |  |
| **H66** | **R** | fragment 1 | GGCCCAGGCGGCC CTG ACT CAG CCG TCC TCG | GCC CTT CAC CGC CGG CCC GTA |
|  |  | fragment 2 | TAC GGG CCG GCG GTG AAG GGC TGT GCC ACC ATC TCG AGG GAC | GGC CGG CCT GGC CAC TAG TGG AGG AGA CGA TGA |
|  |  | overlap | GGCCCAGGCGGCC CTG ACT CAG CCG TCC TCG | GGC CGG CCT GGC CAC TAG TGG AGG AGA CGA TGA |
| **H67** | **A** | fragment 1 | GGCCCAGGCGGCC CTG ACT CAG CCG TCC TCG | ACG GCC CTT CAC CGC CGG CCC |
|  |  | fragment 2 | GGG CCG GCG GTG AAG GGC CGT TGT ACC ATC TCG AGG GAC AAC | GGC CGG CCT GGC CAC TAG TGG AGG AGA CGA TGA |
|  |  | overlap | GGCCCAGGCGGCC CTG ACT CAG CCG TCC TCG | GGC CGG CCT GGC CAC TAG TGG AGG AGA CGA TGA |
| **H68** | **T** | fragment 1 | GGCCCAGGCGGCC CTG ACT CAG CCG TCC TCG | GGC ACG GCC CTT CAC CGC CGG |
|  |  | fragment 2 | CCG GCG GTG AAG GGC CGT GCC TGT ATC TCG AGG GAC AAC GGG | GGC CGG CCT GGC CAC TAG TGG AGG AGA CGA TGA |
|  |  | overlap | GGCCCAGGCGGCC CTG ACT CAG CCG TCC TCG | GGC CGG CCT GGC CAC TAG TGG AGG AGA CGA TGA |
| **H69** | **I** | fragment 1 | GGCCCAGGCGGCC CTG ACT CAG CCG TCC TCG | GGT GGC ACG GCC CTT CAC CGC |
|  |  | fragment 2 | GCG GTG AAG GGC CGT GCC ACC TGT TCG AGG GAC AAC GGG CAG | GGC CGG CCT GGC CAC TAG TGG AGG AGA CGA TGA |
|  |  | overlap | GGCCCAGGCGGCC CTG ACT CAG CCG TCC TCG | GGC CGG CCT GGC CAC TAG TGG AGG AGA CGA TGA |
| **H70** | **S** | fragment 1 | GGCCCAGGCGGCC CTG ACT CAG CCG TCC TCG | GAT GGT GGC ACG GCC CTT CAC |
|  |  | fragment 2 | GTG AAG GGC CGT GCC ACC ATC TGT AGG GAC AAC GGG CAG AGC | GGC CGG CCT GGC CAC TAG TGG AGG AGA CGA TGA |
|  |  | overlap | GGCCCAGGCGGCC CTG ACT CAG CCG TCC TCG | GGC CGG CCT GGC CAC TAG TGG AGG AGA CGA TGA |
| **H71** | **R** | fragment 1 | GGCCCAGGCGGCC CTG ACT CAG CCG TCC TCG | CGA GAT GGT GGC ACG GCC CTT |
|  |  | fragment 2 | AAG GGC CGT GCC ACC ATC TCG TGT GAC AAC GGG CAG AGC ACA | GGC CGG CCT GGC CAC TAG TGG AGG AGA CGA TGA |
|  |  | overlap | GGCCCAGGCGGCC CTG ACT CAG CCG TCC TCG | GGC CGG CCT GGC CAC TAG TGG AGG AGA CGA TGA |
| **H72** | **D** | fragment 1 | GGCCCAGGCGGCC CTG ACT CAG CCG TCC TCG | CCT CGA GAT GGT GGC ACG GCC |
|  |  | fragment 2 | GGC CGT GCC ACC ATC TCG AGG TGT AAC GGG CAG AGC ACA GTG | GGC CGG CCT GGC CAC TAG TGG AGG AGA CGA TGA |
|  |  | overlap | GGCCCAGGCGGCC CTG ACT CAG CCG TCC TCG | GGC CGG CCT GGC CAC TAG TGG AGG AGA CGA TGA |
| **H73** | **N** | fragment 1 | GGCCCAGGCGGCC CTG ACT CAG CCG TCC TCG | GTC CCT CGA GAT GGT GGC ACG |
|  |  | fragment 2 | CGT GCC ACC ATC TCG AGG GAC TGT GGG CAG AGC ACA GTG AGG | GGC CGG CCT GGC CAC TAG TGG AGG AGA CGA TGA |
|  |  | overlap | GGCCCAGGCGGCC CTG ACT CAG CCG TCC TCG | GGC CGG CCT GGC CAC TAG TGG AGG AGA CGA TGA |
| **H74** | **G** | fragment 1 | GGCCCAGGCGGCC CTG ACT CAG CCG TCC TCG | GTT GTC CCT CGA GAT GGT GGC |
|  |  | fragment 2 | GCC ACC ATC TCG AGG GAC AAC TGT CAG AGC ACA GTG AGG CTG | GGC CGG CCT GGC CAC TAG TGG AGG AGA CGA TGA |
|  |  | overlap | GGCCCAGGCGGCC CTG ACT CAG CCG TCC TCG | GGC CGG CCT GGC CAC TAG TGG AGG AGA CGA TGA |
| **H75** | **Q** | fragment 1 | GGCCCAGGCGGCC CTG ACT CAG CCG TCC TCG | CCC GTT GTC CCT CGA GAT GGT |
|  |  | fragment 2 | ACC ATC TCG AGG GAC AAC GGG TGT AGC ACA GTG AGG CTG CAG | GGC CGG CCT GGC CAC TAG TGG AGG AGA CGA TGA |
|  |  | overlap | GGCCCAGGCGGCC CTG ACT CAG CCG TCC TCG | GGC CGG CCT GGC CAC TAG TGG AGG AGA CGA TGA |
| **H76** | **S** | fragment 1 | GGCCCAGGCGGCC CTG ACT CAG CCG TCC TCG | CTG CCC GTT GTC CCT CGA GAT |
|  |  | fragment 2 | ATC TCG AGG GAC AAC GGG CAG TGT ACA GTG AGG CTG CAG CTG | GGC CGG CCT GGC CAC TAG TGG AGG AGA CGA TGA |
|  |  | overlap | GGCCCAGGCGGCC CTG ACT CAG CCG TCC TCG | GGC CGG CCT GGC CAC TAG TGG AGG AGA CGA TGA |
| **H77** | **T** | fragment 1 | GGCCCAGGCGGCC CTG ACT CAG CCG TCC TCG | GCT CTG CCC GTT GTC CCT CGA |
|  |  | fragment 2 | TCG AGG GAC AAC GGG CAG AGC TGT GTG AGG CTG CAG CTG AAC | GGC CGG CCT GGC CAC TAG TGG AGG AGA CGA TGA |
|  |  | overlap | GGCCCAGGCGGCC CTG ACT CAG CCG TCC TCG | GGC CGG CCT GGC CAC TAG TGG AGG AGA CGA TGA |
| **H78** | **V** | fragment 1 | GGCCCAGGCGGCC CTG ACT CAG CCG TCC TCG | TGT GCT CTG CCC GTT GTC CCT |
|  |  | fragment 2 | AGG GAC AAC GGG CAG AGC ACA TGT AGG CTG CAG CTG AAC AAC | GGC CGG CCT GGC CAC TAG TGG AGG AGA CGA TGA |
|  |  | overlap | GGCCCAGGCGGCC CTG ACT CAG CCG TCC TCG | GGC CGG CCT GGC CAC TAG TGG AGG AGA CGA TGA |
| **H79** | **R** | fragment 1 | GGCCCAGGCGGCC CTG ACT CAG CCG TCC TCG | CAC TGT GCT CTG CCC GTT GTC |
|  |  | fragment 2 | GAC AAC GGG CAG AGC ACA GTG TGT CTG CAG CTG AAC AAC CTC | GGC CGG CCT GGC CAC TAG TGG AGG AGA CGA TGA |
|  |  | overlap | GGCCCAGGCGGCC CTG ACT CAG CCG TCC TCG | GGC CGG CCT GGC CAC TAG TGG AGG AGA CGA TGA |
| **H80** | **L** | fragment 1 | GGCCCAGGCGGCC CTG ACT CAG CCG TCC TCG | CCT CAC TGT GCT CTG CCC GTT |
|  |  | fragment 2 | AAC GGG CAG AGC ACA GTG AGG TGT CAG CTG AAC AAC CTC AGG | GGC CGG CCT GGC CAC TAG TGG AGG AGA CGA TGA |
|  |  | overlap | GGCCCAGGCGGCC CTG ACT CAG CCG TCC TCG | GGC CGG CCT GGC CAC TAG TGG AGG AGA CGA TGA |
| **H81** | **Q** | fragment 1 | GGCCCAGGCGGCC CTG ACT CAG CCG TCC TCG | CAG CCT CAC TGT GCT CTG CCC |
|  |  | fragment 2 | GGG CAG AGC ACA GTG AGG CTG TGT CTG AAC AAC CTC AGG GCT | GGC CGG CCT GGC CAC TAG TGG AGG AGA CGA TGA |
|  |  | overlap | GGCCCAGGCGGCC CTG ACT CAG CCG TCC TCG | GGC CGG CCT GGC CAC TAG TGG AGG AGA CGA TGA |
| **H82** | **L** | fragment 1 | GGCCCAGGCGGCC CTG ACT CAG CCG TCC TCG | CTG CAG CCT CAC TGT GCT CTG |
|  |  | fragment 2 | CAG AGC ACA GTG AGG CTG CAG TGT AAC AAC CTC AGG GCT GAG | GGC CGG CCT GGC CAC TAG TGG AGG AGA CGA TGA |
|  |  | overlap | GGCCCAGGCGGCC CTG ACT CAG CCG TCC TCG | GGC CGG CCT GGC CAC TAG TGG AGG AGA CGA TGA |
| **H82A** | **N** | fragment 1 | GGCCCAGGCGGCC CTG ACT CAG CCG TCC TCG | CAG CTG CAG CCT CAC TGT GCT |
|  |  | fragment 2 | AGC ACA GTG AGG CTG CAG CTG TGT AAC CTC AGG GCT GAG GAC | GGC CGG CCT GGC CAC TAG TGG AGG AGA CGA TGA |
|  |  | overlap | GGCCCAGGCGGCC CTG ACT CAG CCG TCC TCG | GGC CGG CCT GGC CAC TAG TGG AGG AGA CGA TGA |
| **H82B** | **N** | fragment 1 | GGCCCAGGCGGCC CTG ACT CAG CCG TCC TCG | GTT CAG CTG CAG CCT CAC TGT |
|  |  | fragment 2 | ACA GTG AGG CTG CAG CTG AAC TGT CTC AGG GCT GAG GAC ACC | GGC CGG CCT GGC CAC TAG TGG AGG AGA CGA TGA |
|  |  | overlap | GGCCCAGGCGGCC CTG ACT CAG CCG TCC TCG | GGC CGG CCT GGC CAC TAG TGG AGG AGA CGA TGA |
| **H82C** | **L** | fragment 1 | GGCCCAGGCGGCC CTG ACT CAG CCG TCC TCG | GTT GTT CAG CTG CAG CCT CAC |
|  |  | fragment 2 | GTG AGG CTG CAG CTG AAC AAC TGT AGG GCT GAG GAC ACC GGC | GGC CGG CCT GGC CAC TAG TGG AGG AGA CGA TGA |
|  |  | overlap | GGCCCAGGCGGCC CTG ACT CAG CCG TCC TCG | GGC CGG CCT GGC CAC TAG TGG AGG AGA CGA TGA |
| **H83** | **R** | fragment 1 | GGCCCAGGCGGCC CTG ACT CAG CCG TCC TCG | GAG GTT GTT CAG CTG CAG CCT |
|  |  | fragment 2 | AGG CTG CAG CTG AAC AAC CTC TGT GCT GAG GAC ACC GGC ACC | GGC CGG CCT GGC CAC TAG TGG AGG AGA CGA TGA |
|  |  | overlap | GGCCCAGGCGGCC CTG ACT CAG CCG TCC TCG | GGC CGG CCT GGC CAC TAG TGG AGG AGA CGA TGA |
| **H84** | **A** | fragment 1 | GGCCCAGGCGGCC CTG ACT CAG CCG TCC TCG | CCT GAG GTT GTT CAG CTG CAG |
|  |  | fragment 2 | CTG CAG CTG AAC AAC CTC AGG TGT GAG GAC ACC GGC ACC TAC | GGC CGG CCT GGC CAC TAG TGG AGG AGA CGA TGA |
|  |  | overlap | GGCCCAGGCGGCC CTG ACT CAG CCG TCC TCG | GGC CGG CCT GGC CAC TAG TGG AGG AGA CGA TGA |
| **H85** | **E** | fragment 1 | GGCCCAGGCGGCC CTG ACT CAG CCG TCC TCG | AGC CCT GAG GTT GTT CAG CTG |
|  |  | fragment 2 | CAG CTG AAC AAC CTC AGG GCT TGT GAC ACC GGC ACC TAC TTC | GGC CGG CCT GGC CAC TAG TGG AGG AGA CGA TGA |
|  |  | overlap | GGCCCAGGCGGCC CTG ACT CAG CCG TCC TCG | GGC CGG CCT GGC CAC TAG TGG AGG AGA CGA TGA |
| **H86** | **D** | fragment 1 | GGCCCAGGCGGCC CTG ACT CAG CCG TCC TCG | CTC AGC CCT GAG GTT GTT CAG |
|  |  | fragment 2 | CTG AAC AAC CTC AGG GCT GAG TGT ACC GGC ACC TAC TTC TGC | GGC CGG CCT GGC CAC TAG TGG AGG AGA CGA TGA |
|  |  | overlap | GGCCCAGGCGGCC CTG ACT CAG CCG TCC TCG | GGC CGG CCT GGC CAC TAG TGG AGG AGA CGA TGA |
| **H87** | **T** | fragment 1 | GGCCCAGGCGGCC CTG ACT CAG CCG TCC TCG | GTC CTC AGC CCT GAG GTT GTT |
|  |  | fragment 2 | AAC AAC CTC AGG GCT GAG GAC TGT GGC ACC TAC TTC TGC GCC | GGC CGG CCT GGC CAC TAG TGG AGG AGA CGA TGA |
|  |  | overlap | GGCCCAGGCGGCC CTG ACT CAG CCG TCC TCG | GGC CGG CCT GGC CAC TAG TGG AGG AGA CGA TGA |
| **H88** | **G** | fragment 1 | GGCCCAGGCGGCC CTG ACT CAG CCG TCC TCG | GGT GTC CTC AGC CCT GAG GTT |
|  |  | fragment 2 | AAC CTC AGG GCT GAG GAC ACC TGT ACC TAC TTC TGC GCC AAA | GGC CGG CCT GGC CAC TAG TGG AGG AGA CGA TGA |
|  |  | overlap | GGCCCAGGCGGCC CTG ACT CAG CCG TCC TCG | GGC CGG CCT GGC CAC TAG TGG AGG AGA CGA TGA |
| **H89** | **T** | fragment 1 | GGCCCAGGCGGCC CTG ACT CAG CCG TCC TCG | GCC GGT GTC CTC AGC CCT GAG |
|  |  | fragment 2 | CTC AGG GCT GAG GAC ACC GGC TGT TAC TTC TGC GCC AAA GGT | GGC CGG CCT GGC CAC TAG TGG AGG AGA CGA TGA |
|  |  | overlap | GGCCCAGGCGGCC CTG ACT CAG CCG TCC TCG | GGC CGG CCT GGC CAC TAG TGG AGG AGA CGA TGA |
| **H90** | **Y** | fragment 1 | GGCCCAGGCGGCC CTG ACT CAG CCG TCC TCG | GGT GCC GGT GTC CTC AGC CCT |
|  |  | fragment 2 | AGG GCT GAG GAC ACC GGC ACC TGT TTC TGC GCC AAA GGT ATT | GGC CGG CCT GGC CAC TAG TGG AGG AGA CGA TGA |
|  |  | overlap | GGCCCAGGCGGCC CTG ACT CAG CCG TCC TCG | GGC CGG CCT GGC CAC TAG TGG AGG AGA CGA TGA |
| **H91** | **F** | fragment 1 | GGCCCAGGCGGCC CTG ACT CAG CCG TCC TCG | GTA GGT GCC GGT GTC CTC AGC |
|  |  | fragment 2 | GCT GAG GAC ACC GGC ACC TAC TGT TGC GCC AAA GGT ATT GAG | GGC CGG CCT GGC CAC TAG TGG AGG AGA CGA TGA |
|  |  | overlap | GGCCCAGGCGGCC CTG ACT CAG CCG TCC TCG | GGC CGG CCT GGC CAC TAG TGG AGG AGA CGA TGA |
| **H93** | **A** | fragment 1 | GGCCCAGGCGGCC CTG ACT CAG CCG TCC TCG | GCA GAA GTA GGT GCC GGT GTC |
|  |  | fragment 2 | GAC ACC GGC ACC TAC TTC TGC TGT AAA GGT ATT GAG AGT GAT | GGC CGG CCT GGC CAC TAG TGG AGG AGA CGA TGA |
|  |  | overlap | GGCCCAGGCGGCC CTG ACT CAG CCG TCC TCG | GGC CGG CCT GGC CAC TAG TGG AGG AGA CGA TGA |
| **H94** | **K** | fragment 1 | GGCCCAGGCGGCC CTG ACT CAG CCG TCC TCG | GGC GCA GAA GTA GGT GCC GGT |
|  |  | fragment 2 | ACC GGC ACC TAC TTC TGC GCC TGT GGT ATT GAG AGT GAT AGT | GGC CGG CCT GGC CAC TAG TGG AGG AGA CGA TGA |
|  |  | overlap | GGCCCAGGCGGCC CTG ACT CAG CCG TCC TCG | GGC CGG CCT GGC CAC TAG TGG AGG AGA CGA TGA |
|  |  |  |  |  |
| **H103** | **W** |  | GGCCCAGGCGGCC CTG ACT CAG CCG TCC TCG | GGCCGGCCTGGCC ACT AGT GGA GGA GAC GAT GAC TTC GGT CCC GTG GCC ACA TGCGTCGATCTCCTC |
| **H104** | **G** |  | GGCCCAGGCGGCC CTG ACT CAG CCG TCC TCG | GGCCGGCCTGGCC ACT AGT GGA GGA GAC GAT GAC TTC GGT CCC GTG ACA CCATGCGTCGATCTC |
| **H105** | **H** |  | GGCCCAGGCGGCC CTG ACT CAG CCG TCC TCG | GGCCGGCCTGGCC ACT AGT GGA GGA GAC GAT GAC TTC GGT CCC ACA GCCCCATGCGTCGAT |
| **H106** | **G** |  | GGCCCAGGCGGCC CTG ACT CAG CCG TCC TCG | GGCCGGCCTGGCC ACT AGT GGA GGA GAC GAT GAC TTC GGT ACA GTGGCCCCATGCGTC |
| **H107** | **T** |  | GGCCCAGGCGGCC CTG ACT CAG CCG TCC TCG | GGCCGGCCTGGCC ACT AGT GGA GGA GAC GAT GAC TTC ACA CCCGTGGCCCCATGC |
| **H108** | **E** |  | GGCCCAGGCGGCC CTG ACT CAG CCG TCC TCG | GGCCGGCCTGGCC ACT AGT GGA GGA GAC GAT GAC ACA GGTCCCGTGGCCCCA |
| **H109** | **V** |  | GGCCCAGGCGGCC CTG ACT CAG CCG TCC TCG | GGCCGGCCTGGCC ACT AGT GGA GGA GAC GAT ACA TTCGGTCCCGTGGCC |
| **H110** | **I** |  | GGCCCAGGCGGCC CTG ACT CAG CCG TCC TCG | GGCCGGCCTGGCC ACT AGT GGA GGA GAC ACA GACTTCGGTCCCGTG |
| **H111** | **V** |  | GGCCCAGGCGGCC CTG ACT CAG CCG TCC TCG | GGCCGGCCTGGCC ACT AGT GGA GGA ACA GATGACTTCGGTCCC |
| **H112** | **S** |  | GGCCCAGGCGGCC CTG ACT CAG CCG TCC TCG | GGCCGGCCTGGCC ACT AGT GGA ACA GACGATGACTTCGGT |
| **H113** | **S** |  | GGCCCAGGCGGCC CTG ACT CAG CCG TCC TCG | GGCCGGCCTGGCC ACT AGT ACA GGAGACGATGACTTC |
| **H114** | **T** |  | GGCCCAGGCGGCC CTG ACT CAG CCG TCC TCG | GGCCGGCCTGGCC ACT ACA GGAGGAGACGATGAC |
| **H115** | **S** |  | GGCCCAGGCGGCC CTG ACT CAG CCG TCC TCG | GGCCGGCCTGGCC ACA AGTGGAGGAGACGAT |
