# Supplementary material for: Chicken scFvs with an Artificial Cysteine for Site-Directed Conjugation
Source: PLoS One. 2016 Jan 14;11(1):e0146907. doi: 10.1371/journal.pone.0146907 (PMC4713166; doi:10.1371/journal.pone.0146907)
Supplement: S2 Table — (DOCX) [file pone.0146907.s004.docx]

**S2 Table. PCR primer list for generation of charge-variant artificial cysteine-mutant**

| **Mutant** | **Fragment** | **Forward (5` -> 3`)** | **Reverse (5` -> 3`)** |
| --- | --- | --- | --- |
| **QCR** | fragment 1 | GGCCCAGGCGGCC CTG ACT CAG CCG TCC TCG | GAG GCC GCC CCC GGA CTC |
|  | fragment2 | GAG TCC GGG GGC GGC CTC CAG TGT CGT GGA GGA GCG CTC AGC | GGC CGG CCT GGC CAC TAG TGG AGG AGA CGA TGA |
|  | overlap | GGCCCAGGCGGCC CTG ACT CAG CCG TCC TCG | GGC CGG CCT GGC CAC TAG TGG AGG AGA CGA TGA |
| **QCK** | fragment 1 | GGCCCAGGCGGCC CTG ACT CAG CCG TCC TCG | GAG GCC GCC CCC GGA CTC |
|  | fragment2 | GAG TCC GGG GGC GGC CTC CAG TGT AAG GGA GGA GCG CTC AGC | GGC CGG CCT GGC CAC TAG TGG AGG AGA CGA TGA |
|  | overlap | GGCCCAGGCGGCC CTG ACT CAG CCG TCC TCG | GGC CGG CCT GGC CAC TAG TGG AGG AGA CGA TGA |
| **RCP** | fragment 1 | GGCCCAGGCGGCC CTG ACT CAG CCG TCC TCG | GAG GCC GCC CCC GGA CTC |
|  | fragment2 | GAG TCC GGG GGC GGC CTC CGT TGT CCC GGA GGA GCG CTC AGC | GGC CGG CCT GGC CAC TAG TGG AGG AGA CGA TGA |
|  | overlap | GGCCCAGGCGGCC CTG ACT CAG CCG TCC TCG | GGC CGG CCT GGC CAC TAG TGG AGG AGA CGA TGA |
| **RCR** | fragment 1 | GGCCCAGGCGGCC CTG ACT CAG CCG TCC TCG | GAG GCC GCC CCC GGA CTC |
|  | fragment2 | GAG TCC GGG GGC GGC CTC CGT TGT CGT GGA GGA GCG CTC AGC | GGC CGG CCT GGC CAC TAG TGG AGG AGA CGA TGA |
|  | overlap | GGCCCAGGCGGCC CTG ACT CAG CCG TCC TCG | GGC CGG CCT GGC CAC TAG TGG AGG AGA CGA TGA |
| **RCK** | fragment 1 | GGCCCAGGCGGCC CTG ACT CAG CCG TCC TCG | GAG GCC GCC CCC GGA CTC |
|  | fragment2 | GAG TCC GGG GGC GGC CTC CGT TGT AAG GGA GGA GCG CTC AGC | GGC CGG CCT GGC CAC TAG TGG AGG AGA CGA TGA |
|  | overlap | GGCCCAGGCGGCC CTG ACT CAG CCG TCC TCG | GGC CGG CCT GGC CAC TAG TGG AGG AGA CGA TGA |
| **KCP** | fragment 1 | GGCCCAGGCGGCC CTG ACT CAG CCG TCC TCG | GAG GCC GCC CCC GGA CTC |
|  | fragment2 | GAG TCC GGG GGC GGC CTC AAG TGT CCC GGA GGA GCG CTC AGC | GGC CGG CCT GGC CAC TAG TGG AGG AGA CGA TGA |
|  | overlap | GGCCCAGGCGGCC CTG ACT CAG CCG TCC TCG | GGC CGG CCT GGC CAC TAG TGG AGG AGA CGA TGA |
| **KCR** | fragment 1 | GGCCCAGGCGGCC CTG ACT CAG CCG TCC TCG | GAG GCC GCC CCC GGA CTC |
|  | fragment2 | GAG TCC GGG GGC GGC CTC AAG TGT CGT GGA GGA GCG CTC AGC | GGC CGG CCT GGC CAC TAG TGG AGG AGA CGA TGA |
|  | overlap | GGCCCAGGCGGCC CTG ACT CAG CCG TCC TCG | GGC CGG CCT GGC CAC TAG TGG AGG AGA CGA TGA |
| **KCK** | fragment 1 | GGCCCAGGCGGCC CTG ACT CAG CCG TCC TCG | GAG GCC GCC CCC GGA CTC |
|  | fragment2 | GAG TCC GGG GGC GGC CTC AAG TGT AAG GGA GGA GCG CTC AGC | GGC CGG CCT GGC CAC TAG TGG AGG AGA CGA TGA |
|  | overlap | GGCCCAGGCGGCC CTG ACT CAG CCG TCC TCG | GGC CGG CCT GGC CAC TAG TGG AGG AGA CGA TGA |
| **QCD** | fragment 1 | GGCCCAGGCGGCC CTG ACT CAG CCG TCC TCG | GAG GCC GCC CCC GGA CTC |
|  | fragment2 | GAG TCC GGG GGC GGC CTC CAG TGT GAT GGA GGA GCG CTC AGC | GGC CGG CCT GGC CAC TAG TGG AGG AGA CGA TGA |
|  | overlap | GGCCCAGGCGGCC CTG ACT CAG CCG TCC TCG | GGC CGG CCT GGC CAC TAG TGG AGG AGA CGA TGA |
| **QCE** | fragment 1 | GGCCCAGGCGGCC CTG ACT CAG CCG TCC TCG | GAG GCC GCC CCC GGA CTC |
|  | fragment2 | GAG TCC GGG GGC GGC CTC CAG TGT GAA GGA GGA GCG CTC AGC | GGC CGG CCT GGC CAC TAG TGG AGG AGA CGA TGA |
|  | overlap | GGCCCAGGCGGCC CTG ACT CAG CCG TCC TCG | GGC CGG CCT GGC CAC TAG TGG AGG AGA CGA TGA |
| **DCP** | fragment 1 | GGCCCAGGCGGCC CTG ACT CAG CCG TCC TCG | GAG GCC GCC CCC GGA CTC |
|  | fragment2 | GAG TCC GGG GGC GGC CTC GAT TGT CCC GGA GGA GCG CTC AGC | GGC CGG CCT GGC CAC TAG TGG AGG AGA CGA TGA |
|  | overlap | GGCCCAGGCGGCC CTG ACT CAG CCG TCC TCG | GGC CGG CCT GGC CAC TAG TGG AGG AGA CGA TGA |
| **DCD** | fragment 1 | GGCCCAGGCGGCC CTG ACT CAG CCG TCC TCG | GAG GCC GCC CCC GGA CTC |
|  | fragment2 | GAG TCC GGG GGC GGC CTC GAT TGT GAT GGA GGA GCG CTC AGC | GGC CGG CCT GGC CAC TAG TGG AGG AGA CGA TGA |
|  | overlap | GGCCCAGGCGGCC CTG ACT CAG CCG TCC TCG | GGC CGG CCT GGC CAC TAG TGG AGG AGA CGA TGA |
| **DCE** | fragment 1 | GGCCCAGGCGGCC CTG ACT CAG CCG TCC TCG | GAG GCC GCC CCC GGA CTC |
|  | fragment2 | GAG TCC GGG GGC GGC CTC GAT TGT GAA GGA GGA GCG CTC AGC | GGC CGG CCT GGC CAC TAG TGG AGG AGA CGA TGA |
|  | overlap | GGCCCAGGCGGCC CTG ACT CAG CCG TCC TCG | GGC CGG CCT GGC CAC TAG TGG AGG AGA CGA TGA |
| **ECP** | fragment 1 | GGCCCAGGCGGCC CTG ACT CAG CCG TCC TCG | GAG GCC GCC CCC GGA CTC |
|  | fragment2 | GAG TCC GGG GGC GGC CTC GAA TGT CCC GGA GGA GCG CTC AGC | GGC CGG CCT GGC CAC TAG TGG AGG AGA CGA TGA |
|  | overlap | GGCCCAGGCGGCC CTG ACT CAG CCG TCC TCG | GGC CGG CCT GGC CAC TAG TGG AGG AGA CGA TGA |
| **ECD** | fragment 1 | GGCCCAGGCGGCC CTG ACT CAG CCG TCC TCG | GAG GCC GCC CCC GGA CTC |
|  | fragment2 | GAG TCC GGG GGC GGC CTC GAA TGT GAT GGA GGA GCG CTC AGC | GGC CGG CCT GGC CAC TAG TGG AGG AGA CGA TGA |
|  | overlap | GGCCCAGGCGGCC CTG ACT CAG CCG TCC TCG | GGC CGG CCT GGC CAC TAG TGG AGG AGA CGA TGA |
| **ECE** | fragment 1 | GGCCCAGGCGGCC CTG ACT CAG CCG TCC TCG | GAG GCC GCC CCC GGA CTC |
|  | fragment2 | GAG TCC GGG GGC GGC CTC GAA TGT GAA GGA GGA GCG CTC AGC | GGC CGG CCT GGC CAC TAG TGG AGG AGA CGA TGA |
|  | overlap | GGCCCAGGCGGCC CTG ACT CAG CCG TCC TCG | GGC CGG CCT GGC CAC TAG TGG AGG AGA CGA TGA |
